# Targeting AXL and RAGE to prevent geminin overexpression-induced triple-negative breast cancer metastasis

**DOI:** 10.1038/s41598-019-55702-w

**Published:** 2019-12-16

**Authors:** Daniel Ryan, Jim Koziol, Wael M. ElShamy

**Affiliations:** 1 Breast Cancer Research Program, San Diego Biomedical Research Institute, San Diego, CA USA; 2 Department of Molecular and Experimental Medicine, The Scripps Research Institute, San Diego, CA USA

**Keywords:** Breast cancer, Breast cancer

## Abstract

Dissemination of metastatic precursors from primaries is the primary reason for patient death. Dissemination encompasses tumor cells invasion of stroma, followed by intravasation through the endothelium barrier into the bloodstream. Here, we describe how geminin-overexpressing tumor cells acquire dissemination ability. Acetylated HMGB1 (Ac-HMGB1) secreted by geminin-overexpressing cells activates RAGE and CXCR4 expression on mesenchymal stem cells (MSCs) located in tumor stroma. Through secreting CXCL12, geminin-overexpressing cells recruit these CXCR4^+^-MSCs into the tumor. Within the tumor, MSCs differentiate into S100A4-secreting cancer-associated fibroblasts (CAFs). S100A4, in a reciprocal manner, activates geminin-overexpressing cells to secrete CCL2 that recruits M0-macrophages from the stroma into the tumor. Within the tumor, CCL2 polarizes M0-macrophages into Gas6-secreting M2-tumor-associated macrophages (M2-TAMs). In concert, geminin-overexpression, S100A4/RAGE and Gas6/AXL signaling promote the invasive and intravasation abilities in geminin-overexpressing cells through exacerbating their stemness and epithelial-to-mesenchymal phenotypes and enhancing expression and functional interaction of CD151 and α3β1-integrin in geminin-overexpressing cells. Tumors formed following injection of geminin-overexpressing cells admixed with MSCs/CAFs grew faster, metastasized earlier, especially to lungs, and were extremely sensitive to anti-c-Abl, anti-RAGE, and anti-AXL drugs. These data support an intrinsic ability in geminin-overexpressing tumor cells to promote their metastatic potential through recruitment and bi-directional interactions with MSCs/CAFs and M2-TAMs.

## Introduction

Geminin is a multifunctional protein that inhibits DNA-replication in S-phase^1^, protect against genomic instability^2^, and safeguard against chemically induced carcinogenesis^3^. Although these functions could support a tumor suppressor function for geminin^2,3^, ablation in mouse embryos or human cells didn′t induce endoreplication or tumorigenesis without stimulus^3,4^. To date, there have never been any reports describing any genetic or epigenetic alterations in the geminin gene in human breast cancer. Our sequencing efforts of the whole gene in ~150 breast tumors confirmed this lack of alterations in geminin gene^4^.

In contrast, geminin is overexpressed in many cancers, including breast cancers, suggesting an oncogenic function instead for geminin^5–9^. Mechanisms to explain this oncogenic role range from ability to maintain pluripotency in early cell lineages^10,11^, promotion of epithelial-to-mesenchymal transition (EMT) in embryonic stem cells^12^, and geminin ability to control proper cytokinesis in G_2_/M/early G_1_-phases^1,4^ in normal human mammary epithelial (HME) cells, while cytokinesis failure and production of aneuploid, aggressive breast cancer cells when overexpressed^13,14^.

In normal cells, during G_2_/M/early G_1_, geminin helps Aurora B chromosome condensation function, and TopoIIα chromosome decatenation function. In breast cancer cells, geminin overexpression blocks Aurora B histone H3-(serine 10) phosphorylation, leading to failure in chromosome condensation, aborted cytokinesis, and aneuploidy^13,14^. In geminin-overexpressing breast cancer cells, TopoIIα is prematurely de-sumoylated and released from chromosomal decatenation sites, also leading to aneuploidy due to elevated chromosomal breakage^14^.

Our recent work showed that, while during S-phase, geminin is a nuclear soluble-serine/threonine (S/T) phosphorylated protein^15^, during G_2_/M/early G_1_-phases, it becomes chromatin bound-tyrosine (Y) phosphorylated protein^4^. While overexpression of wild-type geminin triggers aneuploidy, a single Y-to-A mutant geminin (contains 3 Y-residues at position: 98, 111, and 150)^4^ triggers apoptosis^13^, suggesting geminin oncogenic function requires simultaneous phosphorylation on all 3Ys, implicating the upstream kinases in this function^13^. Our intense search for these kinases revealed the non-receptor tyrosine kinase, c-Abl phosphorylates geminin-Y150 *in vitro*, and *in vivo*^4,13,16^. Indeed, imatinib and nilotinib (two c-Abl inhibitors) promote geminin-overexpressing cell death, *in vitro*, and geminin-overexpressing tumor regression, *in vivo*^16^. Immunohistochemical analysis of several large human breast tumor cohorts revealed geminin overexpression in ~50% of the tumors, while c-Abl overexpression in >90% of the tumors^16^. These analyses also revealed geminin-negative tumors (i.e., expressing normal tissue level), overexpress cytoplasmic c-Abl, while geminin-overexpressing tumors (especially TNBCs) overexpress nuclear c-Abl^16^. We now refer to geminin/nuclear c-Abl co-overexpressing tumors as “GemOE” tumors.

High-mobility group box 1 (HMGB1) is a ubiquitous DNA-binding protein with essential DNA metabolism functions^17^. HMGB1 can be released passively from necrotic cells or actively from activated immune cells, hypoxic, or inflamed cancer cells^18^. HMGB1 secretion requires hyper-acetylation on the chromatin^10,19^. Recently, we described how geminin helps acetylate chromatic HMGB1 and release it from GemOE tumor cells^10^, where it through binding to receptor for advanced glycation end products (RAGE) on GemOE tumor cells activates NF-κB-induced survival, especially those exposed to the harsh condition of hypoxia and inflammation^10^ within the tumor core (*aka* “aggressiveness niche”^20^). Binding of extracellular Ac-HMGB1 to RAGE on naïve mesenchymal stem cells (MSCs) activates NF-κB signaling-induced CXCR4 expression. CXCR4-expressing MSCs are then recruited to CXCL12/SDF1-secreting GemOE cells, *in vitro*, and into the aggressiveness niche, *in vivo*^10,20^.

We expand these data, here, and show MSCs activated by Ac-HMGB1 secrete the calcium-binding protein, S100A4 (*aka* metastasin)^21–24^, a known promoter of breast cancer proliferation, invasion, and metastasis^24–26^. In, TNBCs, expression of a nuclear/cytoplasmic S100A4 is associated with high histological tumor grade and inferior metastasis-free and overall survival^24,27^. We show S100A4 entrains GemOE cells to recruit macrophages into the aggressiveness niche and polarizes them to Gas6-secreting M2-TAMs. GemOE tumor cells overexpress the tyrosine kinase receptor, AXL, that binds Gas6^28^. AXL is overexpressed in breast cancers^29–32^ (especially ERα-negative tumors^29,33^). Activation of AXL and RAGE in GemOE tumor cells converts them into metastatic precursors capable of dissemination from primary tumors through exacerbating the stemness and EMT phenotypes^31^ in them, and the expression and functional interaction of the intravasation-inducing CD151 and α3β1-integrin^34^.

## Results

### GemOE cells recruit and activate MSCs into S100A4-secreting CAFs

**Figure 1:**
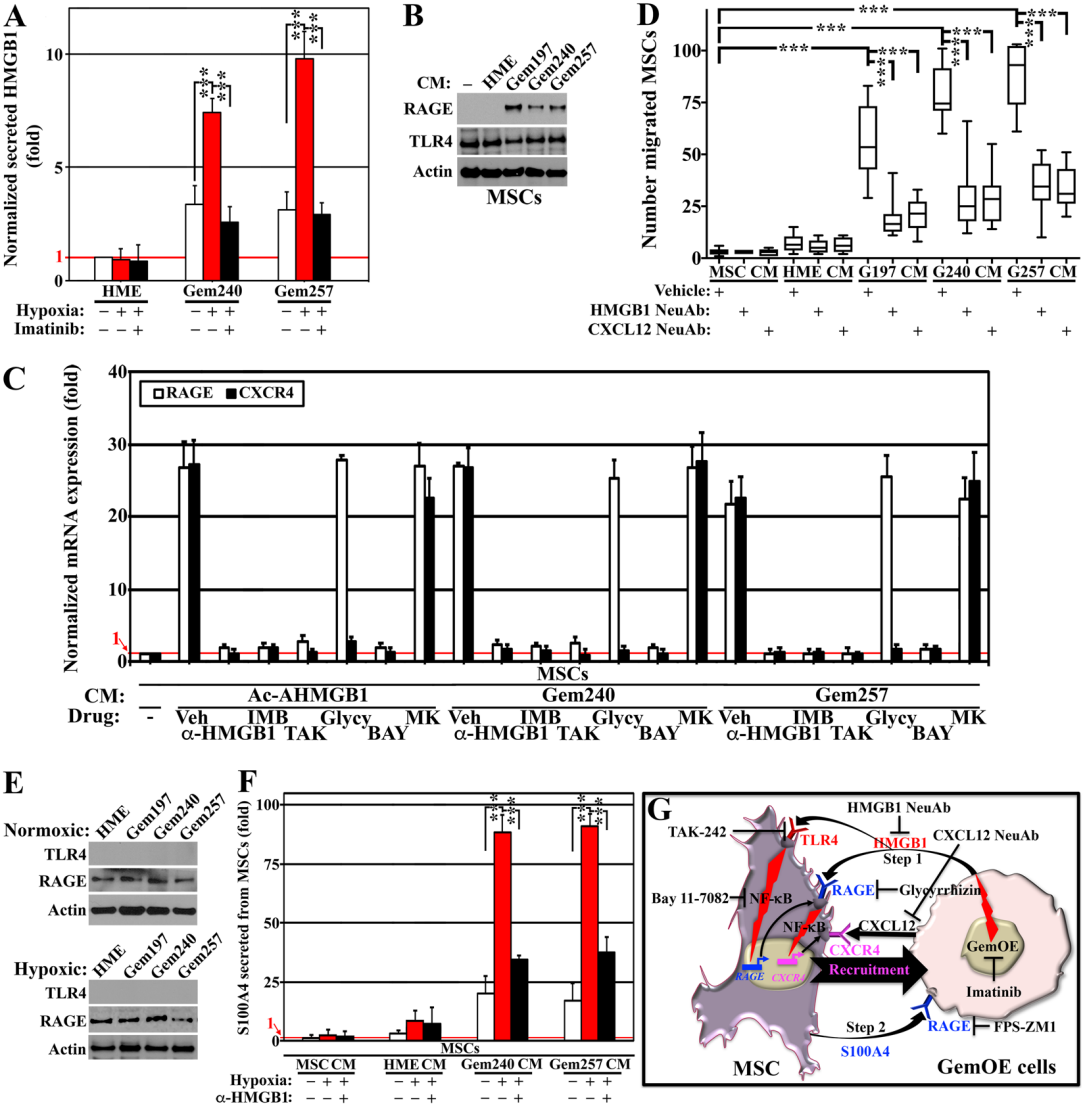
GemOE cells recruit and activate MSCs. (**A**) The level of HMGB1 secreted from the indicated cell lines under normoxic or hypoxic conditions in the absence or presence of imatinib. Assay performed 3 separate times, each in triplicates. (**B**) The levels of RAGE and TLR4 in MSCs exposed to MSCs [−] or indicated cell lines CM for 24 h. The blot was repeated 3 separate times. (**C**) Real-time RT/PCR analysis of *RAGE mRNA* and *CXCR4 mRNA* in MSCs 24 h following exposure to Ac-rHMGB1 or CM from Dox-induced Gem240 or Gem257 cells supplemented with the vehicles, HMGB1 NeuAb, imatinib, TAK-242, glycyrrhizin, BAY 11 7082 or MK-2206. Assay performed 3 separate times, each in triplicates. (**D**) The effect of the indicated cells CM on the migration of MSCs performed for 24 h in Boyden chambers in the presence of the vehicle, HMGB1 or CXCL12 NeuAb. Assay performed 3 separate times, each in triplicates. (**E**) The levels of RAGE and TLR4 in the indicated cell lines exposed 24 h to normoxic (upper) or hypoxic (lower). The blot was repeated 3 separate times. (**F**) The level of S100A4 secreted from MSCs exposed 24 h to indicated cell lines CM under normoxic or hypoxic conditions in the absence or presence of HMGB1 NeuAb. Assay performed 3 separate times, each in triplicates. (**G**) Schematic representation showing the data discussed in the Figure.

Extracellular Ac-HMGB1 activation of RAGE on naïve MSCs triggers CXCR4 expression and recruitment towards CXCL12-secreting GemOE cells^10^. To expand these data, normal HME, or two of the 1° orthotopic GemOE mammary tumors; Gem240, and Gem257 cells were grown (24 h) under normoxia (20% O_2_) or hypoxia (1% O_2_) in Dox-containing media in the presence or absence of imatinib^4,16^. ELISA revealed that compared to CM from cells expressing low-level geminin, induced Gem240 and Gem257 cells CM contained ~3-fold higher HMGB1 (Fig. 1A, white, and compare white to blue, Suppl. Fig. 1). Hypoxia did not affect normal HME or Dox-uninduced cells (Fig. 1, red, and compare blue and black, Suppl. Fig. 1), while exacerbated HMGB1 secretion from Dox-induced cells (Fig. 1A, red, and Suppl. Fig. 1). Imatinib blocked hypoxia-induced effects (compare black to red, Fig. 1A). One-way ANOVA, followed by post hoc Bonferroni tests, confirmed these data (Suppl. Fig. 2).

Naïve MSCs (see [−], Fig. 1B) are RAGE-negative^35,36^, remain negative after exposure to HME cells CM (Fig. 1B). In contrast, exposure (24 h) to Dox-induced Gem197, Gem240, or Gem257 cells CM induced RAGE expression on MSCs surface (blot for membrane proteins, Fig. 1B). In contrast, whether naïve or exposed to CM from any of these cell lines, MSCs express equally high levels of TLR4 (a second HMGB1 receptor^37,38^) on their surface (Fig. 1B).

RNAs were isolated from naïve MSCs exposed (24 h) to Ac-rHMGB1 (10 µg/ml), or CM from Dox-induced Gem240 or Gem257 cells in the presence of vehicles (IgG isotype + DMSO), HMGB1 neutralizing antibody (NeuAb, 10 µg/ml), imatinib, TLR4 TAK-242, glycyrrhizin, Bay 11-7082, or MK-2206. RT/qPCR showed *RAGE mRNA* increases ~30fold in naïve MSCs exposed to Ac-rHMGB1, or CM from Dox-induced Gem240 or Gem257 cells (compare [−] to vehicles, white, Fig. 1C). All drugs except glycyrrhizin and MK-2206 blocked this increase (white bars, Fig. 1C). Moreover, previously, we showed that CXCR4 expression increases in naïve MSCs by Ac-HMGB1 downstream of RAGE^10^. *CXCR4 mRNA* level was also increased by ~30fold in naïve MSCs exposed to Ac-rHMGB1 or CM from Dox-induced Gem240 cells or Gem257 cells (compare [−] to vehicle, black bars, Fig. 1C). All drugs except MK-2206 blocked this increase in *CXCR4 mRNA* expression (black bars, Fig. 1C). Together reinforce our previous published data^10^ and expand them to show Ac-HMGB1 activates TLR4 first to trigger RAGE expression, which then also through activation by Ac-HMGB1 triggers CXCR4 expression. Noteworthy, Ac-HMGB1 in both situations activated NF-κB rather than AKT signaling in MSCs^10^.

Naïve MSCs layered in inserts of Boyden chambers efficiently migrated towards Dox-induced Gem197, Gem240, or Gem257 cells CM not MSCs CM or HME cells CM added in the lower chamber within 24 h (Fig. 1D). The recruitment was almost completely blocked in the presence of HMGB1 or CXCL12 NeuAbs (Fig. 1D). Together, reinforce that a primary function for RAGE/CXCR4 activation in naïve MSCs is to promote their  migration towards GemOE tumor cells (step 1, Fig. 1G).

Unlike naïve MSCs (Fig. 1B), HME and Dox-induced GemOE cells express no TLR4 while expressing equally high-levels of RAGE on their surface (Fig. 1E, upper). Hypoxia did not alter this pattern (Fig. 1E, lower). RAGE also binds the S100 family members^23,24,27,39^. Since RAGE is constitutively expressed on GemOE cells, we evaluated whether activated MSCs express/release HMGB1 or S100 proteins.

RNAs were isolated from naïve MSCs exposed (24 h) to naïve MSCs CM, normal HME cells CM, Dox-induced Gem240, or Gem257 cells CM. RT/qPCR showed that only exposure to Dox-induced Gem240 or Gem257 cells CM induced expression of *S100A2, 4, 6, 8*, and 9, not *S*1*00A1, 7*, or *HMGB1 mRNAs* in naïve MSCs (Suppl. Fig. 3A). Because of S100A4 role in breast cancer metastasis^23–26,40^, we pursued it further. We found that HMGB1 NeuAb blocked the elevation of *S*10*0A4 mRNAs* expression in MSCs following exposure to Dox-induced Gem240 or Gem257 cells CM (Suppl. Fig. 3B).

Moreover, ELISA showed that neither normal HME cells nor Dox-induced GemOE cells secrete S100A4 (not shown). In contrast, naïve MSCs exposed (24 h) to Dox-induced Gem240 or Gem257 cells CM only secrete high-levels of S100A4 (Fig. 1F, white bars). This secretion increased further when hypoxic Dox-induced Gem240 or Gem257 cells CM was used instead (Fig. 1F, red) and almost completely blocked by HMGB1 NeuAb (Fig. 1F, black). One-way ANOVA, followed by post hoc Bonferroni tests, confirmed these data (Suppl. Fig. 4). Together suggest that in the vicinity of GemOE cells, MSCs differentiate into S100A4-secreting cancer-associated fibroblasts^10,41^ (i.e., CAFs, Fig. 1G, step 2).

### S100A4-activated GemOE cells attract and polarize macrophages into Gas6-secreting M2-TAMs

Recently, we showed that IRISOE cells secrete high-levels of CCL2, *in vitro*, and *in vivo* to recruit macrophages^42^. To define whether bidirectional interaction between GemOE cells and MSCs through S100A4 exists was studied next.

**Figure 2:**
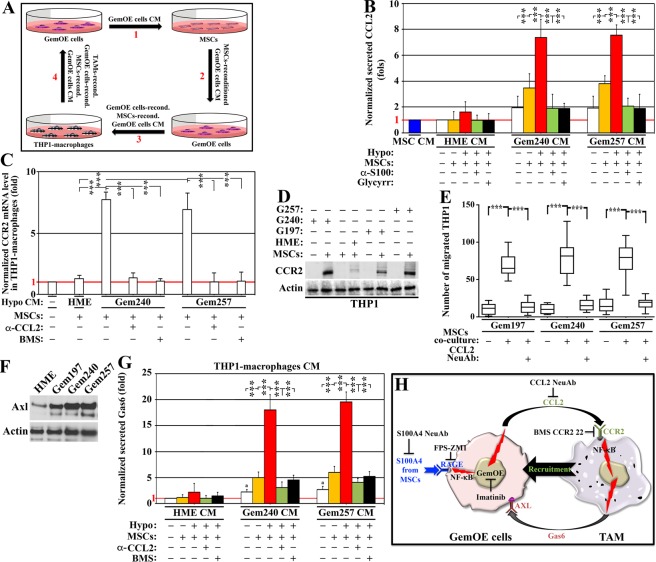
MSCs-reactivated GemOE cells recruit and activate macrophages. (**A**) Schematic representation of the co-culture experiments performed in this study. (**B**) The level of CCL2 secreted from the indicated cell lines under normoxic or hypoxic condition co-cultures with MSCs in the absence or presence of S100A4 NeuAb or glycyrrhizin. Assay performed 3 separate times, each in triplicates. (**C**) Real-time RT/PCR analysis of the *CCR2 mRNA* in THP1-macrophages exposed 24 h to the co-cultures of the indicated cell lines with MSCs supplemented with CCL2 NeuAb or BMS CCR2 22. Assay performed 3 separate times, each in triplicates. (**D**) The expression of CCR2 protein in the indicated cell lines alone or co-cultured with MSC for 24 h. The blot was repeated 3 separate times. (**E**) The effect of the CM from the indicated co-cultures with MSCs for 24 on the migration of THP1-macrophages performed for 24 h in Boyden chambers in the presence of vehicle or CCL2 NeuAb. Assay performed 3 separate times, each in triplicates. (**F**) The levels of AXL in the indicated cells. The blot was repeated 3 separate times. (**G**) The level of Gas6 secreted from THP1-macrophages exposed 24 h to the indicated co-cultures generated under normoxic or hypoxic conditions in the absence or presence of CCL2 NeuAb or BMS CC2 22. Assay performed 3 separate times, each in triplicates. (**H**) Schematic representation showing the data discussed in the Figure.

CM from normal HME cells or Dox-induced Gem240 or Gem257 cells exposed to normoxia or hypoxia (24 h) re-conditioned by exposure to MSCs (another 24 h, Fig. 2A_1_) were re-added to the same cell line in the absence or presence of S100A4 NeuAb or glycyrrhizin (24 h, Fig. 2A_2_). ELISA showed MSCs (Fig. 2B, blue), and normal HME cells exposed to their normoxic or hypoxic CM re-conditioned by MSCs (Fig. 2B, orange and red, respectively) or not (Fig. 2B, white) did not secrete CCL2. In contrast, Dox-induced Gem240 or Gem257 cells secreted low-levels of CCL2 when exposed to their CM (Fig. 2B, white), slightly increased following exposure to their normoxic CM re-conditioned by MSCs (Fig. 2B, orange), and increased even further when exposed to their hypoxic CM reconditioned by MSCs (Fig. 2B, red). Importantly, S100A4 NeuAb (Fig. 2B, green) and glycyrrhizin (Fig. 2B, black) both blocked these increases. One-way ANOVA, followed by post hoc Bonferroni tests, confirmed these data (Suppl. Fig. 5).

The receptor for CCL2, CCR2, controls the trafficking of monocytes into damaged tissues or tumors^42,43^. Recently, we showed CCL2 upregulates CCR2 expression on unpolarized macrophages^42^. PMA-treated THP1-macrophages (hereafter THP1s) were exposed (24 h) to HME cells/or Dox-induced Gem240 or Gem257 cells/MSCs co-cultures CM (Fig. 2A_3_). Real-time RT/qPCR and western blot analysis of these THP1s showed low-levels *CCR2 mRNA* in unexposed THP1s ([−], Fig. 2C). While MSCs only CM did not induce *CCR2 mRNA* and protein expression, a slight increase was detected in THP1s exposed to hypoxic HME cells/MSCs co-culture CM (Fig. 2C,D). In contrast, high-levels *CCR2 mRNA* was detected in THP1s exposed to hypoxic Dox-induced Gem240 or Gem257 cells/MSCs co-culture CM (Fig. 2C). This induction was significantly blocked by CCL2 NeuAb and BMS CCR2 22 (Fig. 2C). Importantly, high-levels CCR2 protein expression was detected in THP1s exposed to Dox-induced Gem197, Gem240, or Gem257 cells/MSCs co-culture CM only (Fig. 2D). Taken together, confirming the role of CCL2 in CCR2 expression in THP1s and primary macrophages^44,45^.

Many THP1s layered in inserts of Boyden chambers migrated 24 h later towards Dox-induced Gem197, Gem240, or Gem257 cells/MSCs co-cultures CM only in the lower chambers. Importantly, in the presence of CCL2 NeuAb, this migration was significantly blocked (Fig. 2E), supporting the CCL2/CCR2 role in macrophages recruitment into GemOE tumors.

Our analysis of receptors upregulated on the surface of the 1° orthotopic GemOE mammary tumors revealed many important metastasis inducing receptors. One such receptor, AXL, was chosen for further analysis in this study due to its pronounced role in intravasation^46^ and breast cancer metastasis^30^. Compared to normal HME cells, Dox-induced Gem197, Gem240, and Gem257 cells express significantly higher levels of AXL on their surfaces (western done on cell membrane extracts, Fig. 2F).

The ligand for AXL; Gas6 also has a notable role in breast cancer progression^28^. Interestingly, compared to normal HME cells CM, Dox-induced Gem240, or Gem257 cells alone CM induced low-levels Gas6 secretion from THP1s (see Fig. 2A_4_,G, white). This secretion was significantly increased when THP1s were instead exposed to normoxic Dox-induced Gem240 or Gem257 cells/MSCs co-culture CM (Fig. 2G, orange). Even higher secretion was detected when hypoxic Dox-induced Gem240 or Gem257 cells/MSCs co-culture CM (Fig. 2G, red). Importantly, CCL2 NeuAb and BMS CCR2 22 significantly blocked Gas6 secretion from THP1s exposed to the hypoxic co-cultures CM (Fig. 2G, green and black, respectively). One-way ANOVA, followed by post hoc Bonferroni tests, confirmed these data (Suppl. Fig. 6). Together suggest S100A4 re-activate GemOE cells to secrete CCL2 that attracts macrophages and polarizes them into Gas6-secreting M2-tumor-associated macrophages (M2-TAMs^42,47,48^, Fig. 2H).

### Imatinib-sensitive MSCs and TAMs recruitment *in vivo*

Previously, we injected shCtrl-, sh-geminin- or shc-Abl-expressing GemOE cells into athymic female mice mammary fat pads. Geminin- or c-Abl-depleted cells developed <20% size tumors, compared to control cells^16^, supporting the intimate relationship between c-Abl and geminin and giving credence to using imatinib to treat GemOE mammary tumors (especially TNBC) see introduction and^16^.

**Figure 3:**
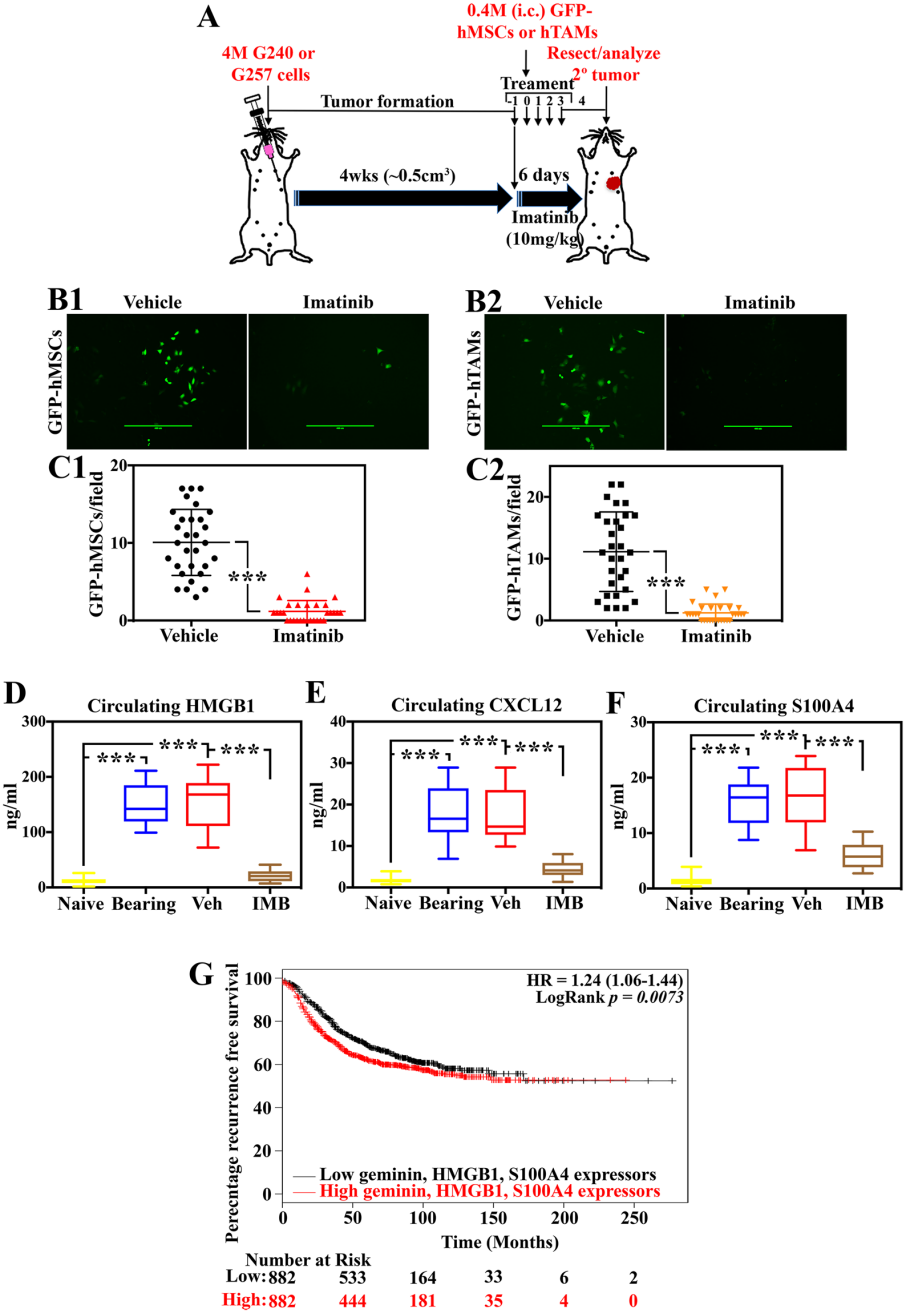
GemOE tumor cells recruit and interact with MSCs and TAMs, *in vivo*. (**A**) Schematic representation of the *in vivo* assay discussed in the Figure. Representative images of GFP-MSCs (**B1**) or GFP-THP1-macrophages (**B2**) found in GemOE tumors treated *in vivo* with vehicle (lefts) or imatinib (rights) according to the protocol in (**A**). The tumors were dissociated into single cells and cultured overnight before photographing. The numbers of GFP-MSCs (**C1**) or GFP-THP1-macrophages (**C2**) found in GemOE tumors treated *in vivo* with vehicle (blacks) or imatinib (red and organ) according to the protocol in (**A**). The tumors were dissociated into single cells and cultured overnight before counting. The levels of HMGB1 (**D**), CXCL12 (**E**), and S100A4 (**F**) in sera isolated from PBs of naïve mice (before tumor cells injection), tumor-bearing mice before or after vehicle or imatinib treatments. (**G**) Kaplan Meir curve of the recurrence-free survival in indicated numbers of patients overexpressing geminin + HMGB1 + S100A4 vs. low expressing patients.

We injected 4 × 10^6^ Gem240 or Gem257 cells in athymic female mice (n = 24/cell line, Fig. 3A), and kept the mice on Dox-supplemented drinking water. Mice developed ~0.5 cm^3^ tumors within 4weeks, at which time (i.e., day -1), mice were randomized into two groups: one received vehicle (DMSO, n = 12/cell line), and the other 10 mg/kg of imatinib (*orally*, n = 12/cell line, Fig. 3A). We activated GFP-human MSCs (GFP-hMSCs) by culturing for a week in CM from Dox-induced Gem240 or Gem257 cells (changed daily), and GFP-THP1s by culturing in Dox-induced Gem240 or Gem257 cells/MSCs co-cultures CM (also changed daily). On day (0), 4 × 10^5^ of activated GFP-hMSCs or GFP-THP1s were intracardiac (*i.c*., through the left ventricle) injected in vehicle (n = 6/cell line) or imatinib (n = 6/cell line) treated mice and treatments were administrated at that time and daily thereafter on days 1, 2, and 3 (Fig. 3A). Tumors and peripheral blood (PB) were collected from all mice on day 4 (Fig. 3A).

Part of each tumor was dissociated into single cells and grew in culture overnight. Next day GFP^+^-cells were counted in at least five high magnification fields (HF) from at least three tumors treated with vehicle or imatinib, and data for both cell lines were pooled (similar results). We detected 10.1 ± 4.2 GFP-hMSCs/HF in cultures from vehicle-treated tumors (Fig. 3B1, left, and 3C1, black) vs. 1.2 ± 1.4 in cultures from imatinib-treated tumors (Fig. 3B1, right and 3C1, red, *p* = *1* × *10*^−6^). Similarly, there was 11.1 ± 6.4 GFP-THP1s/HF in cultures from vehicle-treated tumors (Fig. 3B2, left and 3C2, black) vs. 1.2 ± 1.6 in cultures from imatinib-treated tumors (Fig. 3B2, right, and 3C2, orange, *p* = 1 × 10^−6^).

ELISA performed on sera isolated from the PBs showed (here too, data for similar treated mice were pooled, similar results) that consistent with others^49–51^ and our previous^10,16^ data, HMGB1 level was 14.9 ± 7.4 ng/ml in sera from naïve mice (n = 48, Fig. 3D, yellow), increased to 154.0 ± 36.3 ng/ml in sera from tumor-bearing mice (n = 48, collected on day (−1), *p* < 1 × 10^−6^, Fig. 3D, blue), remained high at 155.2 ± 42.9 ng/ml in sera from vehicle-treated mice (n = 24, collected at euthanasia, *p* < 1 × 10^−6^, Fig. 3D, red), dropped to 24.8 ± 12.5 ng/ml in sera from imatinib-treated mice (n = 24, *p* < 1 × 10^−6^, Fig. 3D, brown). One-way ANOVA, followed by post hoc Bonferroni tests, confirmed these data (Suppl. Fig. 7).

Similarly, CXCL12 level was 1.8 ± 9.7 ng/ml in sera from naïve mice (Fig. 3E, yellow), increased to 18.4 ± 6.4 ng/ml in tumor-bearing mice (*p* < 1 × 10^−6^, Fig. 3E, blue), remained high at 17.1 ± 6.3 ng/ml in vehicle-treated mice (*p* < 1 × 10^−6^, Fig. 3E, red), dropped to 4.1 ± 2.1 ng/ml in imatinib-treated mice (*p* < 1 × 10^−6^, Fig. 3E, brown). One-way ANOVA, followed by post hoc Bonferroni tests, confirmed these data (Suppl. Fig. 8).

Finally, S100A4 level was 1.4 ± 0.8 ng/ml in sera from naïve mice (Fig. 3F, yellow), increased to 15.5 ± 3.7 ng/ml in tumor-bearing mice (*p* < 1 × 10^*−6*^, Fig. 3F, blue), remained high at 16.3 ± 5.1 ng/ml in vehicle-treated mice (*p* < 1 × 10^−6^, Fig. 3F, red), dropped to 6.3 ± 2.7 ng/ml in imatinib-treated mice (*p* < 1 × 10^*−6*^, Fig. 3F, brown). One-way ANOVA, followed by post hoc Bonferroni tests, confirmed these data (Suppl. Fig. 9). Interestingly, analysis of TCGA data confirmed that compared to low geminin + HMGB1 + S100A4-expressing patients, patients expressing high levels had an inferior recurrence-free survival (RFS, *p* = *0.007*, compare red to black, Fig. 3G).

### Further evidence for the recruitment of MSCs and TAMs into GemOE tumors

**Figure 4:**
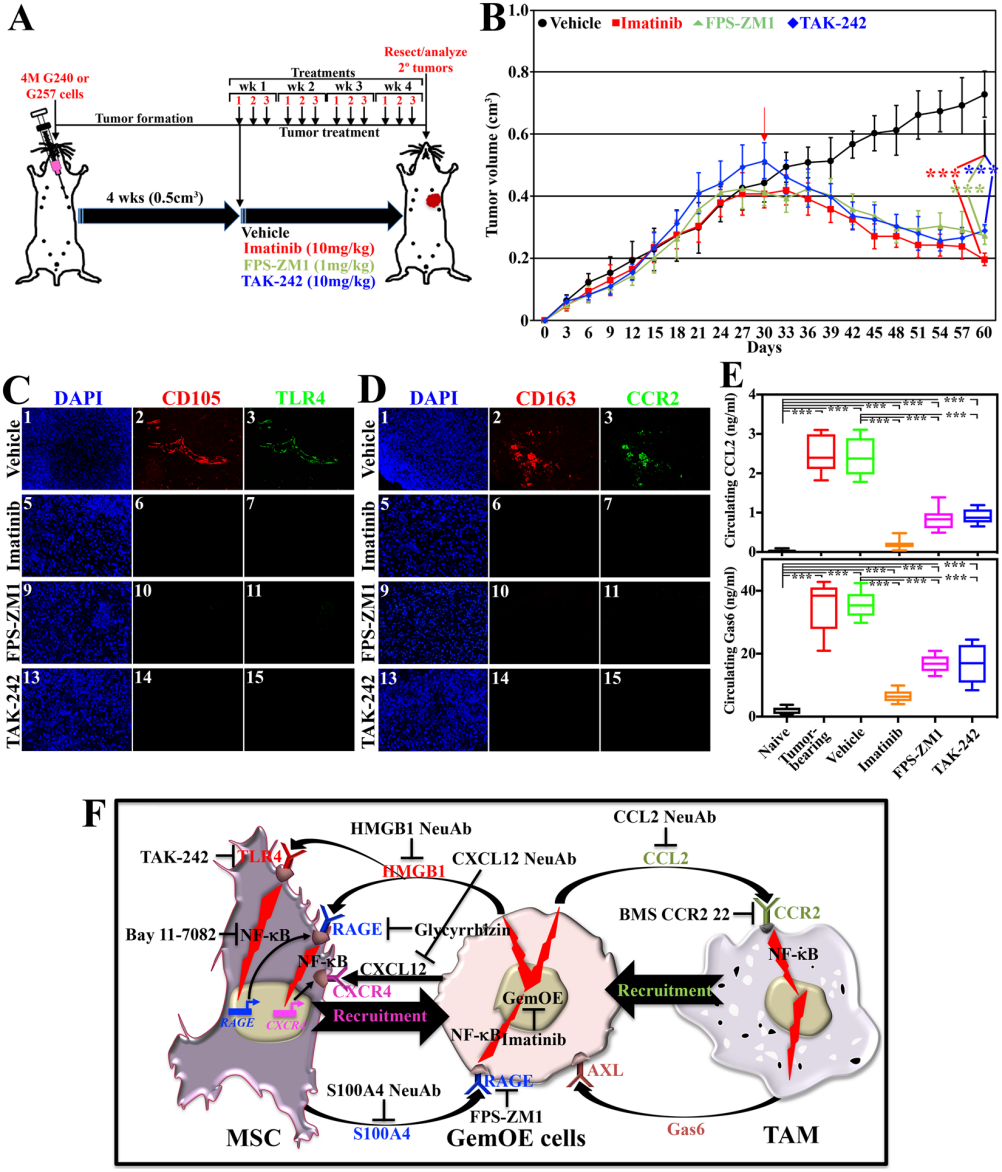
Bi-directional interaction of GemOE cells  with stromal cells in GemOE tumors. (**A**) Schematic representation of the *in vivo* assay discussed in the Figure. (**B**) Gem240 tumor volume (Gem257 cells produced identical data) following the protocol described in (**A**) in the presence of the indicated inhibitors. The red arrow indicates the start of treatments. Student t-test was used to compare vehicle-treated tumors and each treatment separately. Fluorescence IHC staining of sections from the tumors above using mouse-specific anti-CD105 (MSCs specific biomarker) and anti-TLR4 (**C**), or mouse-specific anti-CD163 (macrophage-specific biomarker) and anti-CCR2 (**D**). (**E**) The level of CCL2 (upper) and Gas6 (lower) in naïve mice, tumor-bearing mice before or after vehicle, imatinib, FPS-ZM1, and TAK-242 treatments at tumor resection. (**F**) Schematic representation showing the data discussed so far.

Again, based on our previous experience with imatinib^16^, and because our model specifically calls for macrophages recruitment as a subsequent step for the MSCs recruitment (Fig. 2H), we aimed at further analyze these interactions through inhibiting the two receptors; RAGE and TLR4 we propose mostly involved in MSCs recruitment. We injected 4 × 10^6^ Gem240, or Gem257 cells in athymic female mice (n = 24/cell line, see Fig. 4A) kept on Dox-supplemented drinking water. The mice developed ~0.5 cm^3^ tumor volume within 4weeks, at which time they were randomized into 4 groups received vehicle (DMSO, n = 6), imatinib (10 mg/kg, n = 6), FPS-ZM1^52^ (10 mg/kg, intraperitoneally [*i.p*.], 1 mg/kg, n = 6), or TAK-242^53^ (10 mg/kg, *i.p*. n = 6). Drugs were administered 3times/week for 4weeks (Fig. 4A).

Vehicle-treated Gem240 (identical results were obtained using Gem257) tumors grow ~200% by day 60 (Fig. 4B, black), while imatinib (Fig. 4B, red), FPS-ZM1 (Fig. 4B, green) or TAK-242 (Fig. 4B, blue) treated tumors regressed by ~50% (*p* < 1 × 10^−6^ for all drugs, Fig. 4B). All tumors were resected, paraffin-embedded, sectioned at 4 µm, and immune-stained. Vehicle-treated tumors only showed high-levels CD105 (mouse MSCs fibrogenic differentiation biomarker^54^)/TLR4 co-stained cells (Fig. 4C_1–3_), and CD163 (M2-macrophage-specific biomarker^55^)/CCR2 co-stained cells (Fig. 4D_1–3_). Imatinib (Fig. 4C_5–7_,D_5–7_), FPS-ZM1 (Fig. 4C_9–11_,D_9–11_), or TAK-242 (Fig. 4C_13–15_,D_13–15_) treated mice were negative for both cells.

Finally, sera were isolated from PBs from these mice at euthanasia. ELISA (data from both cell lines were pooled) showed 0.052 ± 0.030 ng/ml of CCL2 in naïve mice sera (n = 48, Fig. 4E, upper-black), increased to 2.358 ± 0.524 ng/ml in tumor-bearing mice sera (n = 48, *p* < 1 × 10^−6^, Fig. 4E, upper-red), remained high at 2.418 ± 0.512 ng/ml in vehicle-treated mice sera (n = 12, *p* < 1 × 10^−6^, Fig. 4E, upper-green). The level dropped to 0.205 ± 0.119 ng/ml in imatinib-treated mice sera (n = 12, *p* < 1 × 10^−6^, Fig. 4E, upper-orange), to 0.756 ± 0.269 ng/ml in FPS-treated mice (n = 12, *p* < 1 *× 10*^−6^, Fig. 4E, upper-pink), and to 0.962 ± 0.182 ng/ml in TAK-242-treated mice (n = 12, *p* < 1 × 10^−6^, Fig. 4E, upper-blue). One-way ANOVA, followed by post hoc Bonferroni tests, confirmed these data (Suppl. Fig. 10).

Similarly, 1.865 ± 0.999 ng/ml of Gas6 was detected in naïve mice sera (n = 48, Fig. 4E, lower-black), increased to 33.534 ± 6.738 ng/ml in tumor-bearing mice sera (n = 48, *p* < 1 × 10^−6^, Fig. 4E, lower-red), remained high at 35.505 ± 4.378 in the vehicle-treated mice sera (n = 12, *p* < 1 × 10^−6^, Fig. 4E, lower-green). The level dropped to 7.073 ± 1.857 ng/ml in imatinib-treated mice sera (n = 12, *p* < 1 × 10^−6^, Fig. 4E, lower-pink), to 9.842 ± 2.256 ng/ml in FPS-treated mice sera (n = 12, *p* < 1 × 10^−6^, Fig. 4E, lower-pink) and to 10.539 ± 2.772 ng/ml in TAK-242-treated mice sera (n = 12, *p* < 1 × 10^−6^, Fig. 4E, lower-blue). One-way ANOVA, followed by post hoc Bonferroni tests, confirmed these data (Suppl. Fig. 11). Together, suggest blocking HMGB1 secretion or function prevents recruitment of MSCs and TAMs into GemOE tumors^10^ and their conversion into pro-tumor S100A4-secreting CAFs and Gas6-secreting M2-TAMs (Fig. 4F) leading to tumor regerssion^56,57^.

### The bi-directional loops are involved in GemOE-induced TNBC metastasis

**Figure 5:**
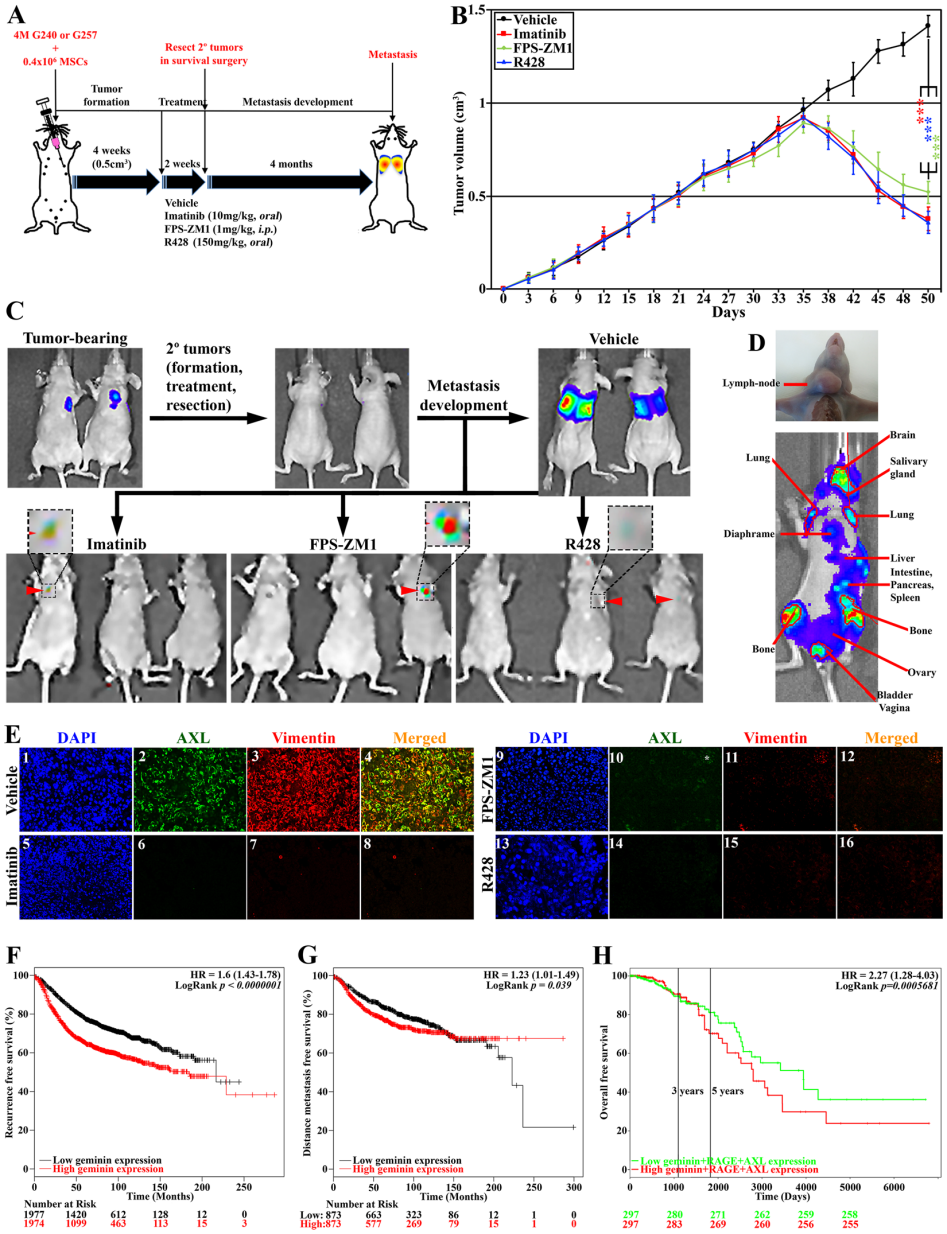
The bi-directional pathways elevate the GemOE tumor metastasis. (**A**) Schematic representation of the *in vivo* assay discussed in the Figure. (**B**) The volume of orthotopic mammary GemOE tumors generated using Gem240 cells. At ~0.5 cm^3^ around week 4, mice were randomized and divided into 4 groups treated with vehicle, imatinib, FPS-ZM1, or R428 daily for 2 weeks (weekend off). Refer to the Results section for more information. Student t-test was used to compare vehicle-treated tumors and each treatment separately. (**C**) Representative images of mice bearing tumors before (upper left), after (upper middle) tumors resection, and macro-metastasis developed within 4 months following vehicle (upper right), not imatinib (lower left), FPS-ZM1 (Lower middle) and R428 (lower right) treatments. Insets show micro-metastasis in the lungs in the treated mice. (**D**) Lymph-node (upper) and widespread (lower) metastases in GemOE tumor-bearing mice. (**E**) Fluorescence IHC staining of sections from the tumors above using anti-AXL and anti-vimentin. Kaplan Meir curves of the recurrence-free survival (**F**) or distant-metastasis-free survival (**G**) of geminin overexpressing patients vs. geminin low expressing patients. (**H**) Kaplan Meir curve of the overall survival of geminin + RAGE + AXL overexpressing patients vs. low expressing patients.

To evaluate HMGB1/S100A4 and CCL2/Gas6 bi-directional loops role in GemOE cells metastatic potential (*cf*. Fig. 4F), we injected 4 × 10^6^ Gem240 or Gem257 cells admixed with 4 × 10^5^ hMSCs in female athymic mice (48mice/cell line, Fig. 5A) kept on Dox-supplemented drinking water. The mice developed ~0.5 cm^3^ tumor volume within 4weeks (Fig. 5A), at which time they were randomized into 4 groups/cell line treated with vehicle (DMSO, n = 12), imatinib (n = 12, 10 mg/kg, *orally*), FPS-ZM1 (n = 12, 1 mg/kg, *i.p*.), or R428 (n = 12, 150 mg/kg, *orally*) daily for 2 weeks (Fig. 5A). Half of the mice were maintained for tumor growth (Fig. 5B). In the other half, tumors were resected in survival surgeries, and metastasis was followed using IVIS for a maximum of 4months (Fig. 5A).

Tumors in vehicle-treated mice increased ~50% within the treatment period (Fig. 5B, black). In contrast, tumors in mice treated with imatinib (Fig. 5B, red), FPS-ZM1 (Fig. 5B, green), or R428 (Fig. 5B, blue) regressed by >50%.

Only in vehicle-treated mice, we detected macro-metastases, especially to the lungs (n = 12, i.e., 6/cell line, Fig. 5C, upper right). Noteworthy, one mouse also developed a lymph-node metastasis (Fig. 5D, upper), and another developed widespread metastasis in almost all organs (Fig. 5D, lower). In contrast, none of the mice injected with either cell line developed metastasis following imatinib (Fig. 5C, lower left), FPS-ZM1 (Fig. 5C, lower middle), or R428 (Fig. 5C, lower right) treatment, although few mice developed very small micro-metastases (arrowheads and insets in Fig. 5C, lower). Detail analysis of these metastases will be reported elsewhere. Importantly, only in vehicle-treated mice, we detected AXL^+^/vimentin^+^ cells in the tumors (compare Fig. 5E_1–4_ to E_5–8_,E_9–12_,E_13–16_).

To support these data further, we carried out Kaplan Meier analysis using several publicly available breast cancer data sets^58–60^. Geminin-overexpressing patients had inferior recurrence-free survival (RFS, n = 3955, HR = 1.6, 95% CI = 1.43–1.78, *p* < 1 × 10^−6^, Fig. 5F) and distance metastasis-free survival (DMFS, n = 1747, HR = 1.23, 95% CI = 1.01–1.49, *p* = *0.039*, Fig. 5G). Geminin + RAGE + AXL-overexpressing patients had inferior overall survival (OS, n = 594, HR = 2.27, 95% CI = 1.28–4.03, *p = 0.0005681*, Fig. 5H), and geminin + HMGB1 + RAGE + CCL2 + AXL-overexpressing patients had inferior lung metastasis-free survival (LMFS, n = 58, HR = 7.35, 95% CI = 2.38–22.63, *p* = *0.0005123*, Suppl. Fig. 12). Together, confirm that positive feedback loops with MSCs/CAFs and/or M2-TAMs, especially within the aggressiveness niche through RAGE and AXL, respectively (*cf*. Fig. 4F) worsen the outcomes for patients with GemOE/TNBC tumors.

### AXL and RAGE enhance GemOE cell invasion ability

HME, Gem197, Gem240, and Gem257 cells were switched to Dox-containing serum-free (SF)-media for 24 h before they were treated in Dox-containing SF-media with none or S100A4 + Gas6 *plus* vehicle, FPS-ZM1, R428, or both for 30 mins or 24 h.

**Figure 6:**
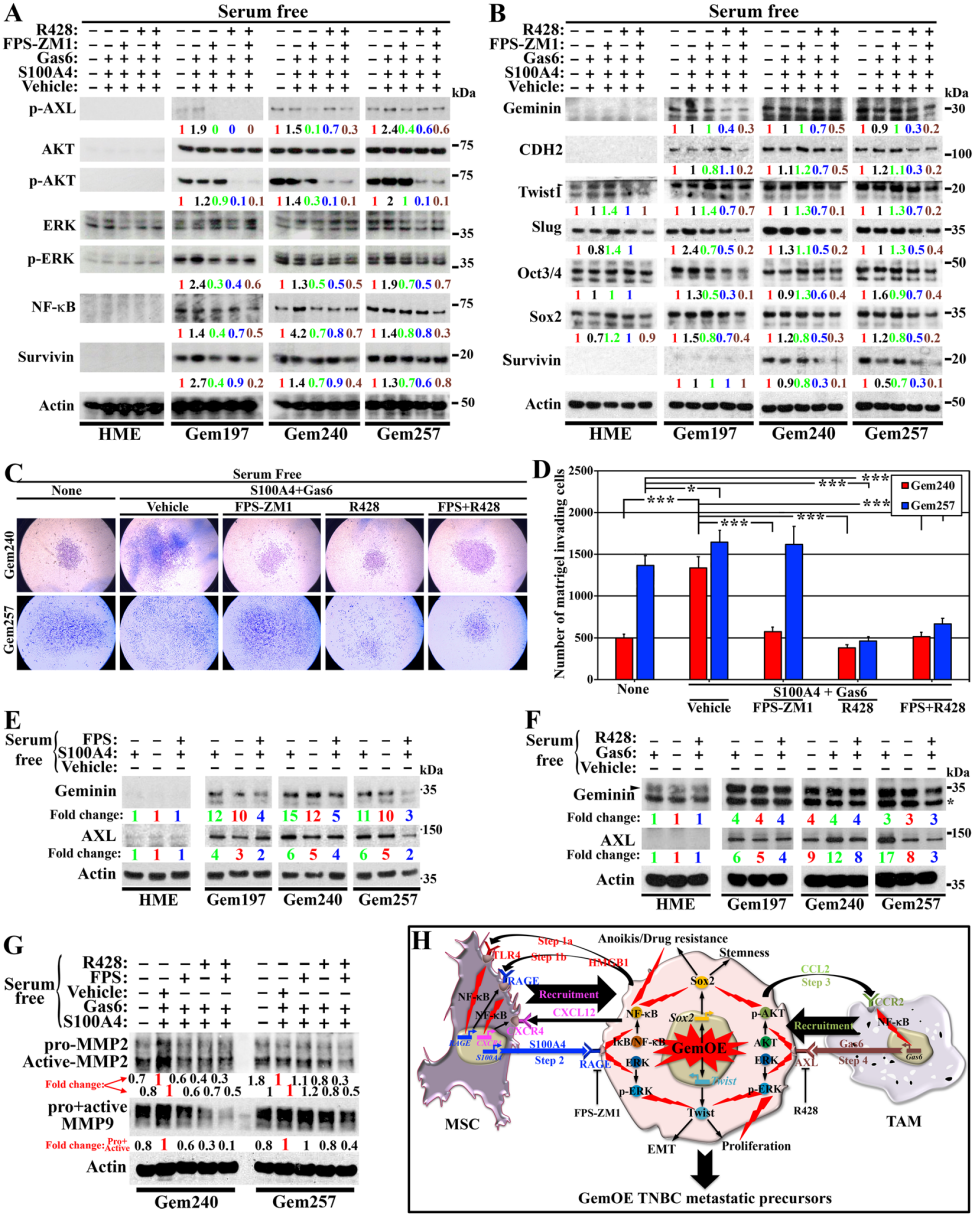
The roles of RAGE and AXL in promoting invasion in GemOE tumor cells. The expression of the indicated proteins in the indicated cell lines following 24 h of growth in Dox-containing SF-media followed by a switch to Dox-containing SF-media supplemented with none, or S100A4 + Gas6 *plus* vehicle, FPS-ZM1, R428, or FPS-ZM1 + R428 for 30 mins (**A**) or 24 h (**B**). (**C**) Representative images of the invasion of Dox-induced Gem240 or Gem257 cells through Matrigel-coated inserts of Boyden chambers 24 h after incubation with SF-media supplemented with none, or S100A4 + Gas6 *plus* vehicle, *plus* FPS-ZM1, *plus* R428, or *plus* FPS-ZM1 + R428. (**D**) Quantitative analysis of the data presented in (**C**). Assay performed 3 separate times, each in triplicates. (**E**) The expression of geminin and AXL in the indicated cell lines following 24 h growth in Dox-containing SF-media followed by switching to Dox-containing SF-media supplemented with none or S100A4 with or without FPS-ZM1. (**F**) The expression of geminin and AXL in the indicated cell lines following 24 h growth in Dox-containing SF- media followed by switching to Dox-containing SF-media supplemented with none or Gas6 with or without R428. (**G**) The expression of MMP2 and MMP9 (pro and active forms) in the indicated cell lines following 24 h growth in Dox-containing SF-media followed by switching to Dox-containing SF-media supplemented with none, or S100A4 + Gas *plus* vehicle, *plus* FPS-ZM1, *plus* R428, or *plus* FPS-ZM1 + R428 for 24 h. (**H**) Schematic representation showing the data discussed in the Figure.

Following 30 min treatments, the membrane/cytoplasmic fractions were isolated. HME cells express no/low-levels of the total (T-)AXL (see below), p-AXL^Y779^, T-AKT, p-AKT^T308/S473^, T-ERK, p-ERK^T183/Y185^, NF-κB and survivin under all conditions (Fig. 6A). In contrast, in SF-media, Dox-induced Gem197, Gem240, or Gem257 cells express >5-fold higher T-AXL (see below), T-AKT, NF-κB, and survivin, and similar levels of T-ERK compared to HME cells (Fig. 6A). Treatment with FPS-ZM1, R428, or both did not significantly affect the expression of total proteins (Fig. 6A). Although the levels of p-AXL^Y779^, p-AKT^T308/S473^, and p-ERK^T183/Y185^ were not significantly affected by S100A4 + Gas6 *plus* vehicle treatment, FPS-ZM1, R428 and even more so in both treatments significantly decreased the levels of these proteins (Fig. 6A), suggesting cross-talk between AXL and RAGE that activates AXL, AKT, and to some extent, ERK signaling in GemOE cells (*cf*. Fig. 6H).

Following the 24 h treatments, cells were sonicated, and whole-cell extracts prepared. HME cells grown in SF-medium expressed low levels of geminin, CDH2, and survivin, while detectable levels of Twist1, Slug, Oct4, and Sox2 (Fig. 6B). In contrast, in SF-media, all Dox-induced GemOE cells expressed high levels of geminin, CDH2, and survivin, and even higher levels of Twist1, Slug, Oct4, and Sox2 compared to HME cells (Fig. 6B). None of the treatments significantly affected the expression of these proteins in HME cells (Fig. 6B), while in SF-media, S100A4 + Gas6 *plus* vehicle elevated the expression of all proteins to different degrees in the three Dox-induced GemOE cell lines (Fig. 6B). In SF-media, S100A4 + Gas6 *plus* FPS-ZM1 significantly decreased the expression of the majority of the proteins except for geminin to different degrees in the three Dox-induced GemOE cell lines (Fig. 6B), *plus* R428 treatment decreased expression of all proteins in some instances marginally, e.g., Twist and Sox2, yet in others significantly, e.g., Slug and Oct4 (Fig. 6B). In SF-media, S100A4 + Gas6 *plus* both drugs treatment, decreased the expression of all proteins even geminin to a level similar to that observed with R428 alone (Fig. 6B), suggesting that RAGE perhaps primes AXL effects.

Next, we layered Gem240 or Gem257 cells in Matrigel-coated inserts of Boyden chambers. In the lower well, we added SF-media containing Dox and none or S100A4 + Gas6 *plus* vehicle, FPS-ZM1, R428, or both. After 24 h, we counted cells invaded the Matrigel and migrated to the other side of the inserts as an *in vitro* confirmation of their stemness and EMT phenotype described above.

Although, many Dox-induced Gem240 and Gem257 cells invaded in SF-condition (Fig. 6C,D), a significant increase in the number of invaded cells was detected in the presence of S100A4 + Gas6 *plus* vehicle (Fig. 6C,D). On the other hand, in the presence of FPS-ZM1 or R428, S100A + Gas6 effect on invasion was significantly blocked (Fig. 6C,D). Again, the reduction in invasion was the same in the presence of both drugs or R428 alone (Fig. 6C,D). One-way ANOVA test followed by post hoc Bonferroni tests confirmed these data (Suppl. Figs. 13 and 14). Together, reinforces that RAGE activation perhaps primes GemOE/TNBC cells to Gas6 effects by affecting geminin and/or AXL expression and/or activity.

To test this hypothesis, we grow HME, Gem197, Gem240, or Gem257 in Dox-containing SF-media for 24 h then switched them to Dox-containing SF-media supplemented with S100A4 *plus* vehicle or FPS-ZM1 (Fig. 6E) or supplemented with Gas6 *plus* vehicle or R428 (Fig. 6F). First, in SF-media, S100A4 elevated geminin and AXL levels in all Dox-induced GemOE (not HME) cells (compare green to red in Fig. 6E). Importantly, the expression of geminin and AXL proteins were significantly decreased in the presence of S100A4 *plus* FPS-ZM1 (compare blue to green in Fig. 6E). It is possible that S100A4/RAGE signaling stabilizes geminin protein (exogenous geminin is expressed from a heterologous promoter) and/or induces its transcription. Geminin, then could affect AXL gene transcription and/or protein stabilization.

Second, in SF-media, Gas6 did not affect geminin expression, while increased AXL expression in all Dox-induced GemOE cells (compare green to red in Fig. 6F). R428 did not affect geminin expression in Dox-induced GemOE cells but inhibited AXL expression (compare blue to green in Fig. 6F). It is possible that S100A4/RAGE signaling separately affects geminin or AXL. Alternatively, positive feedback between geminin and AXL is also possible. We favor the latter since only in Dox-induced GemOE cells, AXL was expressed (Fig. 6E,F).

Finally, in SF-media, Dox-induced Gem240 and Gem257 cells express relatively high levels of pro-/active-MMP2 and -MMP9. Expression of both significantly increased in the presence of S100A4 + Gas6 *plus* vehicle (Fig. 6G) and was blocked in the presence of FPS-ZM1, R428, or both drugs (Fig. 6G). The bidirectional interactions with MSCs/CAFs and M2-TAMs through S100A4/RAGE and Gas6/AXL signaling that activated AKT, ERK, and NF-κB in GemOE cells elevate geminin and/or AXL expression triggering TNBC cells stemness, EMT, and their invasion ability (*cf*. Fig. 6H).

### AXL and RAGE enhance GemOE cells’ intravasation ability

The integrin α3 and β1 (hereafter α3β1-integrin) complex with CD151 (expressed specifically on intravasating tumor cells) is essential to initiate and maintains a tight interaction with the extracellular matrices (ECMs); e.g., laminin I (LMN I), collagen IV (COL IV) and fibronectin (FN) on the basal side of endothelial cells of the vessel^61–63^. To study this in our system, we grew Gem240 or Gem257 for 24 h on uncoated wells or wells that were coated with LMN I, COL IV, or FN in SF-media containing Dox and supplemented with none or S100A4 + Gas6 *plus* vehicle, FPS-ZM1, R428 or both. The next day, wells were washed 3 times, and the cells remaining attached to each well were counted in HF and blotted.

**Figure 7:**
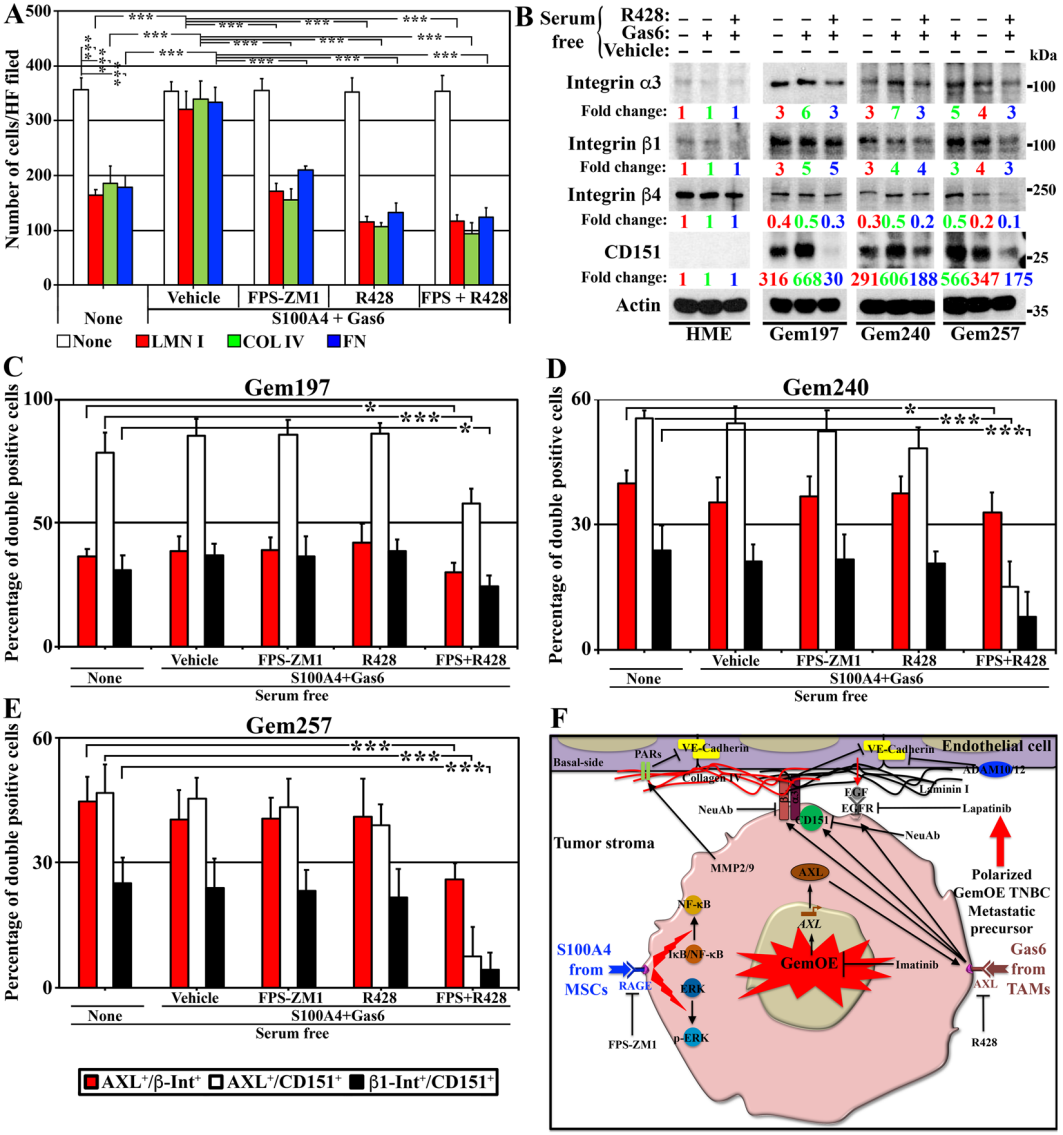
The roles of RAGE and AXL in promoting intravasation in GemOE tumor cells. (**A**) The number of Gem240 cells (Gem257 cells showed identical results) attached to non-coated or LMN I-, COL IV-, or FN-coated wells 24 h after platting followed by washing and counting of attached cells in at least 4 HPFs. Each assay was performed 3 separate times, each in triplicates. (**B**) The expression of the indicated proteins in the indicated cell lines following 24 h growth in Dox-containing SF-media followed by a switch to Dox-containing SF-media supplemented with none, or Gas6 *plus* vehicle, or *plus* R428 for 24 h. Note, the actin blot is the same as in Fig. 6F because it is from the same experiment. Percentage of AXL^+^/β1-integrin^+^ populations (red), the AXL^+^/CD151^+^ populations (white), or β1-integrin^+^/CD151^+^ populations (black) derived from FACS analysis of non-permeabilized Dox-induced Gem197 (**C**), Gem240 (**D**), and Gem257 (**E**) treated as indicated. Each assay was performed 3 separate times, each in triplicates. (**F**) Schematic representation of the data in the entire study as well as future directions.

The Gem240 cells (identical results were obtained with Gem257 cells, not shown) showed a high binding ability to uncoated wells under all conditions (white, Fig. 7A). In comparison, binding to LMN I, COL IV, and FN was significantly decreased under SF conditions (none, Fig. 7A). The binding to all matrices was restored in SF containing S100A4 + Gas6 *plus* vehicle (Fig. 7A), which was blocked by FPS-ZM1, R428, or both treatments (Fig. 7A). One-way ANOVA, followed by post hoc Bonferroni tests, confirmed these data (Suppl. Fig. 15). Together, suggest that AXL and/or RAGE positively affect the binding of intravasating tumor cells with the ECM on the vessels within GemOE tumors.

To study the contribution of CD151 and α3β1 in this binding, we again in SF-media grow normal HME cells, or Dox-induced Gem197, Gem240, or Gem257 cells in the presence of none, or Gas6 *plus* vehicle or *plus* R428. HME expressed very low levels of α3-integrin, β1-integrin, while a high level of β4-integrin (β4) and no CD151 (Fig. 7B). In contrast, in SF-media, all Dox-induced GemOE cell lines expressed high levels of α3-integrin and β1-integrin, low levels of β4-integrin, and very high levels of CD151 (red in Fig. 7B). Gas6 treatment did not affect the expression of these proteins in normal HME cells, but significantly increased expression of α3-integrin, β1-integrin and CD151 in all Dox-induced GemOE cells by >2fold (compare green to red in Fig. 7B). Importantly, inactivating AXL significantly blocked Gas6-induced expression of all four proteins in all Dox-induced GemOE cell lines (compare blue to green in Fig. 7B), suggesting a direct role for AXL in GemOE intravasation ability.

To establish this further, Gem197, Gem240, and Gem257 cells (HME cells were omitted from this analysis since they did not express CD151) grown in Dox-containing SF-media supplemented with none or A4 + Gas6 *plus* vehicle, FPS-ZM1, R428 or both (24 h). Non-permeabilized cells were first stained with FITC-anti-CD44, PE-anti-CD24. The stem-like (i.e., CD44^+^/CD24^−^) population within each cell line was FACS sorted out and stained with PE-Cy7-anti-AXL, 561 620_20-anti-β1-integrin, and APC-anti-CD151 for FACS analysis.

#### AXL^+^/β1^+^ cell populations

All cell lines contained ~40% of AXL^+^/β1^+^ cell populations in SF-media (red, Fig. 7C–E), that didn’t change in the presence of S100A4 + Gas6 *plus* vehicle in any of the cell lines (red, Fig. 7C–E). Although, S100A4 + Gas6 *plus* FPS-ZM1 or *plus* R428 didn’t change this population in any of the cell lines, in S100A4 + Gas6 *plus* both a slightly yet significantly decreased in the AXL^+^/β1^+^-populations in all cell lines was detected (red, Fig. 7C–E).

#### AXL^+^/CD151^+^ cell populations

All cell lines contained >60% of AXL^+^/CD151^+^ cell populations in SF-media (white, Fig. 7C–E), that didn’t change in S100A4 + Gas6 *plus* vehicle, *plus* FPS-ZM1 or *plus* R428 treatments (white, Fig. 7C–E). Importantly, a significant decrease in the percentage of the AXL^+^/CD151^+^ population was observed in all cell lines following S100A4 + Gas6 *plus* both drug treatment (Fig. 7C–E).

#### β1-integrin^+^/CD151^+^ cell populations

All cell lines contained 25–40% of β1^+^/CD151^+^ cell populations in SF-media (black, Fig. 7C–E). Again, this percentage was not significantly changed in S100A4 + Gas6 *plus* vehicle, *plus* FPS-ZM1 or *plus* R428 treatments (black, Fig. 7C–E). Importantly, a significant decrease in the percentage of this β1^+^/CD151^+^ cell populations was observed in all cell lines following treatment with S100A4 + Gas6 *plus* both drugs (black, Fig. 7C–E). Taken together, we propose that GemOE triggers expression and/or activation of AXL and RAGE through interactions with the microenvironment that, in turn, enhances the expression and the functional interaction between α3β1-integrin and CD151. This complex by tightly binding to the ECM on the tumor side of endothelial cells enhances the intravasation ability of GemOE/TNBC metastatic precursors through the endothelium barrier upon dissemination (*cf*. Fig. 7F).

## Discussion

The vast majority of breast cancer deaths are due to metastatic diseases. Inflammation and hypoxia within the tumor microenvironment, especially the core (i.e., the aggressiveness niche^20^) exacerbate metastasis. Here, we elucidated the mechanistic role of the bi-directional interactions between GemOE tumor cells and the microenvironment in promoting GemOE metastatic precursors invasion and intravasation abilities.

MSCs and TAMs are recruited into tumors through tumor secreted factors. MSCs are recruited by Ac-HMGB1/CXCL12^27,64^, while TAMs by CCL2^31,65^. We resolved an issue that was unclear in our previous study^10^, how Ac-HMGB1 induced CXCR4 expression through RAGE in the RAGE-negative naïve MSCs? We found that Ac-HMGB1 activates TLR4 first, which then upregulates RAGE expression in naïve MSCs^66–68^ (*cf*. Fig. 1G). Earlier reports also showed that HMGB1 promotes CXCR4 expression through RAGE and TLR4^10,69^.

Interestingly, in our experiments, Ac-HMGB1 activated NF-κB rather than AKT signaling via TLR4. A recent study showed that lipopolysaccharide (LPS) signaling activates AKT via TLR4 to promote breast cancer metastasis^66^. It is possible that during breast cancer metastasis, Ac-HMGB1 and LPS each initiate a specific signaling pathway downstream of TLR4^70^.

Another important aspect of our studies is that extracellular S100A4 instigates a tumor-supportive microenvironment in GemOE/TNBC tumors, as was recently shown^71^. High S100A4 expression associates with poor outcome in early-stage cancers^24^ and direct interaction between S100A4 and RAGE was recently shown to promote motility in colorectal cancer cells via ERK-dependent mechanism^72,73^, and prostate cancer cells via NF-κB-activated pathway^40^. Chronic inflammation enhances RAGE expression in many cancers^25,72,74,75^, which was directly linked to low-rate patient survival. In our current analysis of a public dataset of >1700 breast cancer samples, low RFS in patients with high geminin + HMGB1 + S100A4 (two RAGE ligands)-expressing patients was observed (Fig. 3H). This observation supports the conclusion that RAGE signaling is involved in enhancing aggressiveness in GemOE TNBC tumors (*cf*. Fig. 3G). We propose that Ac-HMGB1 plays a previously unappreciated role in early-stage TNBCs.

TAMs presence within TNBC tumors is recognized as a critical factor in tumor progression and could be a prognostic factor of a worse outcome^49,50,56,57^. Most human tumors exhibit TAMs with an M2-like phenotype involved in promoting EMT and contributing to tumor progression and drug resistance^49–51^. In keeping, the presence of a high number of CD163^+^-M2 macrophages^76^ in GemOE orthotopic TNBC tumors was correlated with aggressive behavior^50^. We propose that TAM secretome, including Gas6, contributes to this aggressiveness through activation of the AXL receptor, which is overexpressed on GemOE/TNBC tumor cells. Indeed, recently published reports^49^ demonstrate that mesenchymal-like breast cancer cells induce the release of Gas6 selectively from M2-type macrophages^49^.

It is possible that along with c-Abl, AXL is an additional therapeutic target to prevent GemOE metastatic precursors dissemination from TNBC primaries. It is possible that *in vivo* selective inhibition of AXL using R428 could cooperate with imatinib to impair GemOE/TNBC cell invasion and intravasation, entrapping these GemOE/TNBC metastatic precursors within primaries to be resected during surgery reducing the metastatic burden. In support of this conclusion, we found that high geminin + AXL expression was associated with reduced RFS, DMFS, OS and LMFS in TNBC patients (*cf*. Fig. 5F–H, and Suppl. Fig. 12)

Our study has some limitations. Although, we provide evidence for intrinsic ability in GemOE/TNBC tumor cells to recruit and activate stromal elements, such as MSCs and TAMs, the specific requirement of RAGE and AXL for the interaction with MSCs and TAMs, and the distinct biological role in different subtypes of the TNBC disease warrant further studies. Despite these limitations, our results suggest that RAGE and AXL are prognostic indicators of outcome in GemOE/TNBCs patients.

Another limitation is the fact that direct testing for S100A4 in TAMs recruitment into GemOE/TNBC tumors should be done, *in vivo*. Although all the drugs used in this study are specific for their cognate target, their effect in our system should be tested in future studies to provide mechanistic insight, for instance, using the same assays except in cell lines harboring knockdown/knockout of these targets. Also, in future assays, we will assess whether the recruitment of M2-macrophages only or the macrophages, in general, is affected by imatinib, for example, using FACS analysis for the CD11b/F4/80 population within GemOE/TNBC tumors.

Moreover, we demonstrated AXL-mediated elevation in CD151, α3, and β1-integrin expression and functional interaction enhance binding to ECM *in vitro*, which could also be the case on vessels within tumors. This observation would be consistent with previous reports suggesting AXL’s role in breast cancer cells intravasation of the endothelium barrier into the bloodstream^46^. Noteworthy here, in preliminary data (not shown), we found that β1-integrin is phosphorylated in GemOE. An exciting possibility is that this phosphorylation is AXL- or AXL/CD151-driven. In support of the former, R428 abolished this phosphorylation event in GemOE cells (not shown). CD151 may also be a target along with c-Abl and AXL for therapy of GemOE/TNBC tumors.

Our analysis showing the opposite effect for GemOE on β4-integrin expression is intriguing. At present, the functional significance of this observation remains unknown. It is possible since α3β4-integrin is also a receptor for ECM binding in breast cancers^77^, that β4 is involved in the extravasation rather than the intravasation step of GemOE/TNBC metastatic spread, as described recently^48^. This will be investigated further in a follow-up study. Another critical experiment that will also be reported in a soon to be published article is the importance of MSCs/CAFs in the proposed intravasation of GemOE/TNBC cells.

These findings implicate synergistic signals simultaneously mediate multiple mechanisms in GemOE/TNBC intravasation. This study provides new understanding of the signaling pathways activated in GemOE/TNBC cells through interactions with stromal cells (MSCs/CAFs and M2-TAMs) that modulate the intravasation ability of tumor cells and underlines the importance of probing the process of tumor dissemination from an essential yet hitherto under-explored bi-directional interactions between GemOE/TNBC tumor cells and their microenvironmental cells. The dependence of tumor intravasation on the microenvironment provides yet another point of intervention to prevent tumor cell dissemination and patients’ demise^78^.

## Methods

### Cell culture

Human MSCs were purchased from Texas A&M HSC COM Institute for Regenerative Medicine, expanded, and frozen down. THP-1 cells were purchased from ATCC (catalog number: TIB-202^TM^), expanded, and frozen down. For both cell types, one vial is usually propagated for ≤5 generations to perform experiments before a new vail is used. Parental HME cell line (i.e., normal HME, used as control through the studies performed here) was transfected with a retrovirus expressing doxycycline (Dox)-inducible geminin allele^14^. Several clones from these cells were generated by antibiotic-selection, expanded, and tested for geminin expression^14^. A clone named Gem9 overexpressing a level of geminin similar to that observed in breast cancer cell lines was selected to use further. Gem9 cells were orthotopically injected in Dox-supplemented female SCID mice mammary fat pads (n = 10) and the primary (1°)-geminin overexpressing tumors developed were collected^13^ and used to generate the 1° geminin-overexpressing tumor cell lines we named; Gem197, Gem240, Gem256, Gem257, and Gem270. These cell lines were described earlier^10^, and are usually maintained in RPMI medium supplemented with 10% FBS, 1% antibiotics, and 2 µg/ml doxycycline to induce geminin expression will be used to perform the assays in this study. All commercial and in-house cell lines were authenticated by STR profiling and tested for mycoplasma contamination.

### Growth factors, cytokines, and drugs

Recombinant (r)S100A4, rGas6, and rHMGB1, were from Sino Biological. Acetylation of rHMGB1 was described earlier^10^. Imatinib, RAGE inhibitor; glycyrrhizin^79,80^, NF-κB inhibitor; Bay 11-7082^81^, or AKT inhibitor; MK-2206^82,83^, RAGE inhibitor; FPS-ZM1, and CCR2 inhibitor; BMS CCR2 22 (Toronto Research Chemicals Inc.), TLR4 inhibitor; TAK-242^68,84^ (TOCRIS), Glycyrrhizic acid ammonium salt (Sigma) and AXL-specific inhibitor, R428 (Cayman Chemical). All were dissolved in DMSO, working concentration = 10 µM.

### Antibodies

Mouse [m]-Twist (ab50887), m-CD105 (ab114052), m-HMGB1 (ab77302), rabbit [r]-Geminin (ab12147), r-TLR4 (ab13556), r-AXL (ab37861), r-RAGE (ab172473), m-α3-integrin (ab8985), m-β1-integrin (ab24693), r-CCR2 (ab32144) were from abcam. R-NF-κB/p65 (sc-372), m-OCT4 (sc-5279), r-ERK1/2 (137F5), r-Slug (#9585), r-Survivin (6E4), goat [g] MMP2 (sc-6838), g-MMP9 (sc-6840), m-p-ERK (sc-7383) were from Santa Cruz. R-SDF1 (#3740), m-Sox2 (#4900), r-AKT1 (C73H1b), r-p-AKT (T308 “C31E5E/S437”/S437 “D9E”) were from Cell signaling. M-HMGB1 (#07-584), m-vimentin (IF01) were from Millipore. PE-CD163 (560933), m-N-Cadherin (610920) were from R&D Systems. M-p-AXL (MAB6965), m-CCL2 (MAB679) were from ThermoFisher. APC-AXL (DS7HAXL) was from BD Biosciences. M-Actin (#cp01) was from Calbiochem. M-CD151 (350405) was from Biolegend. M-PE-Vio770-CD151 (130-103-728) was from Miltenyi. M-β4-integrin (MCA1456T) was from BIO-RAD. R-S100A4 (GTX62977) was from GeneTax. All antibodies were tested before use.

### Serum isolation from mouse blood

Serum over plasma was chosen to study because it is more representative of inflammation. Samples were collected in a covered test tube and allowed to clot undisturbed at RT for ~30 min. The clot was then removed by centrifuging at 1,000–2,000 g for 10 min at 4 °C. The isolated serum is immediately transferred into a clean polypropylene tube aliquoted into 0.5 µl aliquots and stored in −80 °C.

### ELISA analysis

Wells of a PVC microtiter plate were coat with the antigen by pipetting 50 μl of the conditioned medium (CM) or Serum (or dilution thereof in PBS) in triplicates and plate covered and incubated for overnight at 4 °C. After washing 3x with PBS, blocking of none-specific sites was done using 5% BSA in PBS incubated for ~2 h at room temperature (RT) followed by 3x washing in PBS. Diluted 1° antibody was added for 2 hours at RT, followed by 3x washing in PBS. HRP-conjugated 2° antibody diluted in blocking buffer was incubated 1 hour at RT then 3x washed with PBS. Detection using OPD **(**o-phenylenediamine dihydrochloride) tables and detection at 492 nm was done.

### Cytokine array

HME and HME/geminin cells were assessed for differential cytokine secretion using human cytokine antibody array (RayBio). Conditioned media from an equal number of HME and HME/geminin cells plated in a serum-free medium for 20 h under standard conditions were used to perform according to the manufacturer’s instructions and previously described^10^.

### Co-culture experiment

Boyden chambers (BD biosciences) of 8 µm (for migration) or 0.4 µm (for secretome) pore size were used. Certain cells (or their CM) were layered in the lower chamber with or without neutralizing antibodies, and test cells were layered in the transwell inserts. Cells migrated to the lower compartment of the Boyden chamber were counted and plotted 24 h later. Occasionally, hypoxia was introduced.

### Conditioned media transfer experiment

Conditioned medium (CM) generated for analysis of secreted cytokines and surface receptor expression under normoxia (20% O_2_ for 24 h), or hypoxia (1% O_2_ for 24 h) conditions from an equal number of cells were transferred onto MSCs in the presence or absence of drugs or NeuAb. MSCs re-CM medium was transferred onto HME or GemOE cells with or without drugs or NeuAb for 24 h to re-reconditioned. The resultant CM was then transferred to an equal number of THP1-macrophages for 24 h to be re-re-reconditioned. Finally, this CM was re-added onto HME or GemOE cells for 24 h. At every step, the receiving cells were seeded at equal numbers to avoid number variations discrepancies. At various points, ELISA on CM and western blot on membrane fractions or whole-cell extracts generated by sonication was done.

### Quantitative Real-time RT/PCR

In brief, 100 ng of total RNA isolated using TRIzol was used for qRT/PCR using iScript^TM^ One-Step RT-PCR kit with SYBR Green (Bio-Rad) as previously described^10^. The primer sequences used in this study are:

*RAGE*: Forward: 5′-GACTCTTAGCTGGCACTTGGAT-3′ and Reverse: 5′-GGACTTCACAGGTCAGGGTTAC-3′^85^,

*CXCR4*: Forward: 5′-TTCTACCCCAATGACTTGTG-3′ and Reverse: 5′-ATGTAGTAAGGCAGCCAACA-3′^86^,

*S100A1*: Forward: 5′-CCATGGAGACCCTCATCAAT-3′ and Reverse: 5′-TTCTGGACATCCAGGAAGC-3′^87^,

*S100A2*: Forward: 5′-GAACTTCTGCACAAGGAGCTG-3′ and Reverse: 5′-GACAGTGATGAGTGCCAGGA-3′,

*S100A4*: Forward: 5′-CCACAAGTACTCGGGCAAAG-3′ and Reverse: 5′-GTCCCTGTTGCTGTCCAAGT-3′^88^,

*S100A6*: Forward: 5′-AAGCTGCAGGATGCTGAAAT-3′ and Reverse: 5′-CCCTTGAGGGCTTCATTGTA-3′^89^,

*S100A7*: Forward: 5′-AGACGTGATGACAAGATTGAC-3′ and Reverse: 5′-TGTCTTTTTTCTCAAAGACGTC-3′^90^,

*S100A8*: Forward: 5′-GCTAGAGACCGAGTGTCCTCAG-3′ and Reverse: 5′-GCCCATCTTTATCACCAGAATG-3′,

*S100A9*: Forward: 5′-TGGAGGACCTGGACACAAATG-3′ and Reverse: 5′-TCGTCACCCTCGTGCATCTT-3′^91^,

*HMGB1*: Forward: 5′-ATATGGCAAAAGCGGACAAG-3′ and Reverse: 5′-AGGCCAGGATGTTCTCCTTT-3′^92^,

*GAPDH*: Forward: 5′-TGCACCACCAACTGCTTAGC-3′ and Reverse: 5′-GGCATGGACTGTGGTCATGAG-3′^93^.

### *In vivo* tumorigenicity assay

The University of Mississippi Medical Center IACUC committees approved all animal experiments. All experiments were performed in accordance with NIH guidelines and regulations. Six- to eight-week-old anesthetized immune-compromised Nu/Nu (Harlan) mice were injected with cells re-suspended in 100 µl RPMI medium and Matrigel (1:1) using a 27-gauge needle orthotopically in the 2^nd^ left mammary gland. Tumors were monitored weekly by *i.p*. injection of 100 µl of D-luciferin (15 mg/mL in PBS) and anesthetized mice using a mix of oxygen and isoflurane gas were photographed for luciferase *in vivo* signals with IVIS machine (Xenogen). Tumor volume was assessed twice weekly by caliper (Life Sciences) using the formula L.W/2 (L = longest and W = shortest diameter of the tumor). Tumor initiation was defined as the time when tumors were 3 mm in diameter. Mice were sacrificed when the tumors reached <1.5 cm^3^ in volume or at the indicated time points in Results. At the end of the experiments, mice were euthanized by compressed 100% CO_2_ gas, tumors and organs (e.g., lung) were resected, weighed, fixed in formalin, and later cut at 4 µm for histological and immunohistochemical analysis. Some tumor parts were flash-frozen for DNA, RNA, and protein preparation later. In some instances, from anesthetized mice (using a mix of oxygen and isoflurane gas), 200 µl of blood was drawn from the heart before they were euthanized.

### *In vivo* drug treatments

Tumor-bearing mice were treated with the drugs, concentration, and routes indicated in Results.

### Preparation and injection of GFP-MSCs or -THP1s

GFP-expressing MSCs or THP1 were generated using lentivirus GFP-expressing plasmid. Antibiotics selected clones were propagated and stored frozen. Tumor-bearing mice and treated as described in Results were intracardiac injected through the left ventricle. Following tumor resection, part of the tumor was dissociated using Collagenase I and the protocol used by^94^, grown in culture for 24 h to adhere, and GFP-cells were photographed, counted, and plotted using Prism 7®.

### Fluorescence IHC

IHC was performed as previously described^95^. Briefly, 5μm thick paraffin-embedded sections of tumor tissue excised from GemOE orthotopic mammary tumors were deparaffinized, rehydrated, and washed in PBS. Antigen retrieval for all antibodies was done using Sodium citrate buffer (10 mM Sodium citrate, 0.05% Tween 20, pH 6.0), 10 min at 95 °C. Slides were blocked with 10% normal serum (2° antibody species) for 1 h at RT, washed, and probed with 1° antibodies overnight at 4 °C. Slides were exposed to Alexa Fluor 568 (red), and 488 (green) conjugated secondary antibody for 1 h at RT and counterstained and mounted with VECTASHIELD mounting medium with DAPI (Vector) and imaged under the fluorescence microscope.

### Overall survival (OS) and replace-free survival (RFS) and metastasis-free survival (MFS) analysis

The association of geminin alone or combined with the other factors was investigated for stratified patient cohorts using overall (OS) and relapse/recurrent- (RFS), or distant metastasis- (MFS), or lung metastasis- (LMFS) -free survival using the PROGgeneV2 - Pan-Cancer Prognostics Database (http://watson.compbio.iupui.edu/chirayu/proggene/database) or the Kaplan-Meier survival analysis.

### Statistical analysis

Comparisons of treatment outcomes were tested for statistical differences using the Student t-test for paired data. Additionally, SPSS software was used to perform the ANOVA test followed by a post hoc Bonferroni correction for comparing multiple groups. The association of mRNA transcript expression with various clinicopathological parameters was also analyzed. Statistical significance was assumed at a P-value are *≤0.05, **≤0.01 and ***≤0.001.

## Supplementary information

Suppl. data set

## Data Availability

The data that support the findings of this study are available from the corresponding author [WeS] upon reasonable request.
