# Supplementary material for: Targeting AXL and RAGE to prevent geminin overexpression-induced triple-negative breast cancer metastasis
Source: Sci Rep. 2019 Dec 16;9:19150. doi: 10.1038/s41598-019-55702-w (PMC6915698; doi:10.1038/s41598-019-55702-w)
Supplement: Supplementary file 1 — Suppl. data set [file 41598_2019_55702_MOESM1_ESM.pdf]

Targeting AXL and RAGE to prevent geminin overexpression-induced triple negative breast cancer metastasis.

Daniel Ryan, Jim Koziol, Wael M. ElShamy

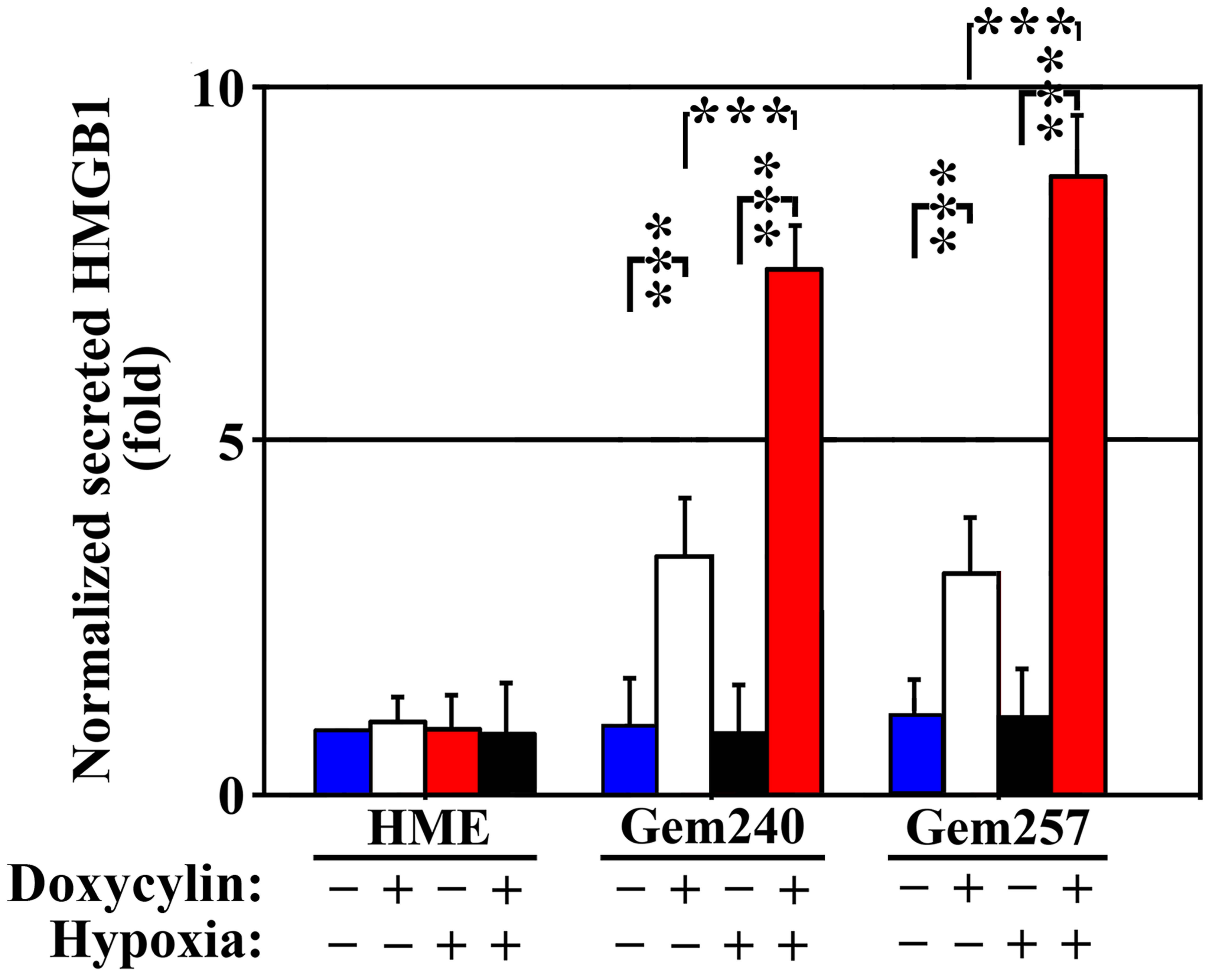
Normalized secreted HMGB1 (fold)
10
5
0
HME
Gem240
Gem257
Doxycylin:
Hypoxia:
− + − + − + − + − + − +
− − + + − − + + − − + +
***
***
***
***
***
***

A

Tests of Between-Subjects Effects

Dependent Variable: HMGB1_ELISA

Source	Type III Sum of Squares	df	Mean Square	F	Sig.	Partial Eta Squared
Corrected Model	1.280E+9 [a]	2	640005024	6.247	.003	.138
Intercept	1.892E+10	1	1.892E+10	184.688	.000	.703
Program	1.280E+9	2	640005024	6.247	.003	.138
Error	7.992E+9	78	102457372			
Total	2.819E+10	81				
Corrected Total	9.272E+9	80				

a. R Squared = .138 (Adjusted R Squared = .116)

B

Post Hoc Tests

Multiple Comparisons

Dependent Variable: HMGB1 ELISA

Bonferroni

(I) Program	(J) Program	Mean Difference (I-J)	Std. Error	Sig.	95% Confidence Interval	
					Lower Bound	Upper Bound
None	Hypoxia	-7155.185*	2754.89291	.034	-13895.598	-414.7719
	H + Imatinib	2142.0370	2754.89291	1.000	-4598.3763	8882.4503
Hypoxia	None	7155.1852*	2754.89291	.034	414.7719	13895.5985
	H + Imatinib	9297.2222*	2754.89291	.003	2556.8089	16037.6355
H + Imatinib	None	-2142.0370	2754.89291	1.000	-8882.4503	4598.3763
	Hypoxia	-9297.222*	2754.89291	.003	-16037.636	-2556.8089

Based on observed means.
The error term is Mean Square(Error) = 102457372.005.

*. The mean difference is significant at the .05 level.

Program(= different treatement)

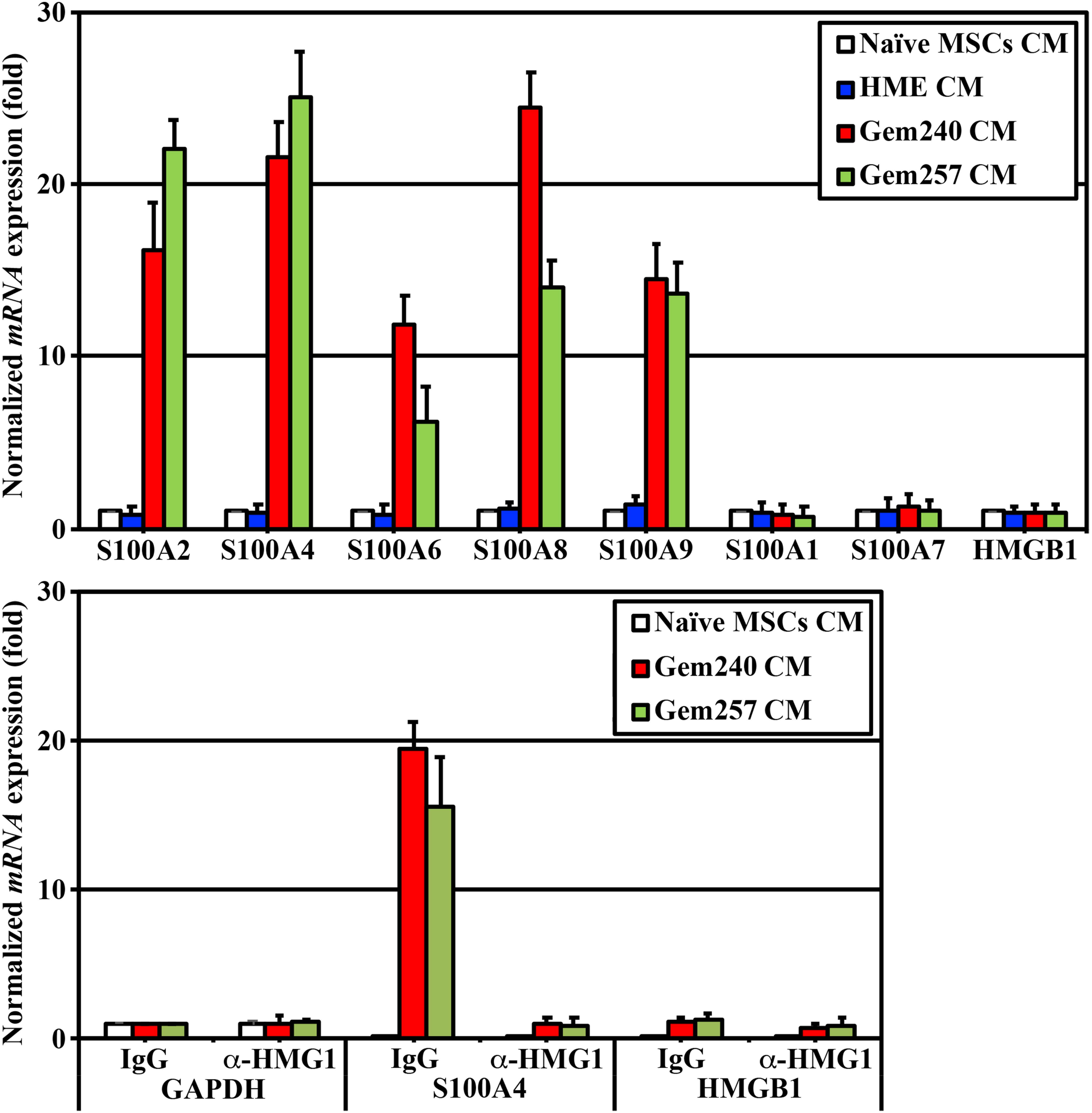
Naïve MSCs CM
HME CM
Gem240 CM
Gem257 CM
Normalized mRNA expression (fold)
0
10
20
30
S100A2
S100A4
S100A6
S100A8
S100A9
S100A1
S100A7
HMGB1
Naïve MSCs CM
Gem240 CM
Gem257 CM
Normalized mRNA expression (fold)
0
10
20
30
IgG
α-HMG1
IgG
α-HMG1
IgG
α-HMG1
GAPDH
S100A4
HMGB1

A

Tests of Between-Subjects Effects

Dependent Variable: S100A4_ELISA

Source	Type III Sum of Squares	df	Mean Square	F	Sig.	Partial Eta Squared
Corrected Model	5.981E+9 [a]	2	2.991E+9	15.325	.000	.226
Intercept	2.354E+10	1	2.354E+10	120.634	.000	.535
Program	5.981E+9	2	2.991E+9	15.325	.000	.226
Error	2.049E+10	105	195157398			
Total	5.002E+10	108				
Corrected Total	2.647E+10	107				

a. R Squared = .226 (Adjusted R Squared = .211)

B

Post Hoc Tests

Multiple Comparisons

Dependent Variable: S100A4 ELISA

Bonferroni

(I) Program	(J) Program	Mean Difference (I-J)	Std. Error	Sig.	95% Confidence Interval Lower Bound	95% Confidence Interval Upper Bound
None	Hypoxia	-18101.11 *	3292.73103	.000	-26112.127	-10090.096
	H+anti-HMGB1	-7183.2500	3292.73103	.094	-15194.266	827.7655
Hypoxia	None	18101.111 *	3292.73103	.000	10090.0956	26112.1266
	H+anti-HMGB1	10917.861 *	3292.73103	.004	2906.8456	18928.8766
H+anti -HMGB1	None	7183.2500	3292.73103	.094	-827.7655	15194.2655
	Hypoxia	-10917.86 *	3292.73103	.004	-18928.877	-2906.8456

Based on observed means.
The error term is Mean Square(Error) = 195157397.626.

*. The mean difference is significant at the .05 level.

Program (= different treatement)

A

Tests of Between-Subjects Effects

Dependent Variable: CCL2_ELISA

Source	Type III Sum of Squares	df	Mean Square	F	Sig.	Partial Eta Squared
Corrected Model	8.775E+9 [a]	4	2.194E+9	34.980	.000	.518
Intercept	2.594E+10	1	2.594E+10	413.571	.000	.761
Program	8.775E+9	4	2.194E+9	34.980	.000	.518
Error	8.153E+9	130	62712636.8			
Total	4.286E+10	135				
Corrected Total	1.693E+10	134				

a. R Squared = .518 (Adjusted R Squared = .504)

B

Post Hoc Tests

Multiple Comparisons

Dependent Variable: CCL2_ELISA

Bonferroni

(I) Program	(J) Program	Mean Difference (I-J)	Std. Error	Sig.	95% Confidence Interval Lower Bound	95% Confidence Interval Upper Bound
1.00	2.00	-5980.5556	2155.31448	.063	-12135.565	174.4534
	3.00	-20585.63*	2155.31448	.000	-26740.639	-14430.621
	4.00	156.6296	2155.31448	1.000	-5998.3794	6311.6386
	5.00	451.0000	2155.31448	1.000	-5704.0090	6606.0090
2.00	1.00	5980.5556	2155.31448	.063	-174.4534	12135.5646
	3.00	-14605.07*	2155.31448	.000	-20760.083	-8450.0651
	4.00	6137.1852	2155.31448	.051	-17.8238	12292.1942
	5.00	6431.5556*	2155.31448	.034	276.5466	12586.5646
3.00	1.00	20585.630*	2155.31448	.000	14430.6206	26740.6386
	2.00	14605.074*	2155.31448	.000	8450.0651	20760.0831
	4.00	20742.259*	2155.31448	.000	14587.2503	26897.2683
	5.00	21036.630*	2155.31448	.000	14881.6206	27191.6386
4.00	1.00	-156.6296	2155.31448	1.000	-6311.6386	5998.3794
	2.00	-6137.1852	2155.31448	.051	-12292.194	17.8238
	3.00	-20742.26*	2155.31448	.000	-26897.268	-14587.250
	5.00	294.3704	2155.31448	1.000	-5860.6386	6449.3794
5.00	1.00	-451.0000	2155.31448	1.000	-6606.0090	5704.0090
	2.00	-6431.556*	2155.31448	.034	-12586.565	-276.5466
	3.00	-21036.63*	2155.31448	.000	-27191.639	-14881.621
	4.00	-294.3704	2155.31448	1.000	-6449.3794	5860.6386

Based on observed means.

The error term is Mean Square(Error) = 62712636.840.

*. The mean difference is significant at the .05 level.

Program (= different treatement)

1.00 = CM alone

2.00 = CM reconditioned with MSCs

3.00 = CM reconditioned with MSCs under hypoxia

4.00 = CM reconditioned with MSCs under hypoxia in the presnece of anti-S100A4 NeuAb

5.00 = CM reconditioned with MSCs under hypoxia in the presnece of Glyzzehherin

A

Tests of Between-Subjects Effects

Dependent Variable: Gas6_ELISA

Source	Type III Sum of Squares	df	Mean Square	F	Sig.	Partial Eta Squared
Corrected Model	1.171E+10 [a]	4	2.928E+9	148.831	.000	.821
Intercept	1.365E+10	1	1.365E+10	693.851	.000	.842
Program	1.171E+10	4	2.928E+9	148.831	.000	.821
Error	2.558E+9	130	19675239.8			
Total	2.792E+10	135				
Corrected Total	1.427E+10	134				

a. R Squared = .821 (Adjusted R Squared = .815)

B

Post Hoc Tests

Multiple Comparisons

Dependent Variable: Gas6_ELISA

Bonferroni

(I) Program	(J) Program	Mean Difference (I-J)	Std. Error	Sig.	95% Confidence Interval Lower Bound	95% Confidence Interval Upper Bound
1.00	2.00	-3059.7778	1207.23866	.124	-6507.3326	387.7770
	3.00	-24916.04*	1207.23866	.000	-28363.592	-21468.482
	4.00	-1111.3704	1207.23866	1.000	-4558.9252	2336.1844
	5.00	-3099.0370	1207.23866	.114	-6546.5918	348.5178
2.00	1.00	3059.7778	1207.23866	.124	-387.7770	6507.3326
	3.00	-21856.26*	1207.23866	.000	-25303.814	-18408.704
	4.00	1948.4074	1207.23866	1.000	-1499.1474	5395.9622
	5.00	-39.2593	1207.23866	1.000	-3486.8141	3408.2955
3.00	1.00	24916.037*	1207.23866	.000	21468.4822	28363.5918
	2.00	21856.259*	1207.23866	.000	18408.7045	25303.8141
	4.00	23804.667*	1207.23866	.000	20357.1119	27252.2215
	5.00	21817.000*	1207.23866	.000	18369.4452	25264.5548
4.00	1.00	1111.3704	1207.23866	1.000	-2336.1844	4558.9252
	2.00	-1948.4074	1207.23866	1.000	-5395.9622	1499.1474
	3.00	-23804.67*	1207.23866	.000	-27252.221	-20357.112
	5.00	-1987.6667	1207.23866	1.000	-5435.2215	1459.8881
5.00	1.00	3099.0370	1207.23866	.114	-348.5178	6546.5918
	2.00	39.2593	1207.23866	1.000	-3408.2955	3486.8141
	3.00	-21817.00*	1207.23866	.000	-25264.555	-18369.445
	4.00	1987.6667	1207.23866	1.000	-1459.8881	5435.2215

Based on observed means.
The error term is Mean Square(Error) = 19675239.807.
*. The mean difference is significant at the .05 level.

Program (= different treatement)

1.00 = THP1 CM reconditioned with mammary cells
2.00 = THP1 CM reconditioned with mammary cells reconditioned with MSCs
3.00 = THP1 CM reconditioned with mammary cells reconditioned with MSCs under hypoxia
4.00 = THP1 CM reconditioned with mammary cells reconditioned with MSCs under hypoxia in the presence of anti-CCL2 NeuAb
5.00 = THP1 CM reconditioned with mammary cells reconditioned with MSCs under hypoxia in the presence of BMS CCR2 22

A

Tests of Between-Subjects Effects

Dependent Variable: HMGB1vivo_ELISA

Source	Type III Sum of Squares	df	Mean Square	F	Sig.	Partial Eta Squared
Corrected Model	437125.61 [a]	3	145708.538	179.152	.000	.854
Intercept	675193.760	1	675193.760	830.168	.000	.900
Program	437125.615	3	145708.538	179.152	.000	.854
Error	74825.625	92	813.322			
Total	1187145.00	96				
Corrected Total	511951.240	95				

a. R Squared = .854 (Adjusted R Squared = .849)

B

Post Hoc Tests

Multiple Comparisons

Dependent Variable: HMGB1vivo_ELISA

Bonferroni

(I) Program	(J) Program	Mean Difference (I-J)	Std. Error	Sig.	95% Confidence Interval Lower Bound	95% Confidence Interval Upper Bound
1.00	2.00	-137.5833*	8.23267	.000	-159.7827	-115.3839
	3.00	-142.0417*	8.23267	.000	-164.2411	-119.8423
	4.00	-10.1667	8.23267	1.000	-32.3661	12.0327
2.00	1.00	137.5833*	8.23267	.000	115.3839	159.7827
	3.00	-4.4583	8.23267	1.000	-26.6577	17.7411
	4.00	127.4167*	8.23267	.000	105.2173	149.6161
3.00	1.00	142.0417*	8.23267	.000	119.8423	164.2411
	2.00	4.4583	8.23267	1.000	-17.7411	26.6577
	4.00	131.8750*	8.23267	.000	109.6756	154.0744
4.00	1.00	10.1667	8.23267	1.000	-12.0327	32.3661
	2.00	-127.4167*	8.23267	.000	-149.6161	-105.2173
	3.00	-131.8750*	8.23267	.000	-154.0744	-109.6756

Based on observed means.
The error term is Mean Square(Error) = 813.322.

*. The mean difference is significant at the .05 level.

Program (= different treatement)

1.00 = Naive mice

2.00 = Tumor-bearing mice

3.00 = Tumor-bearing mice + Vehicle

4.00 = Tumor-bearing mice + Imatinib

A

Tests of Between-Subjects Effects

Dependent Variable: CXCL12vivo_ELISA

Source	Type III Sum of Squares	df	Mean Square	F	Sig.	Partial Eta Squared
Corrected Model	5421.191 [a]	3	1807.064	85.675	.000	.736
Intercept	10622.191	1	10622.191	503.610	.000	.846
Program	5421.191	3	1807.064	85.675	.000	.736
Error	1940.471	92	21.092			
Total	17983.853	96				
Corrected Total	7361.662	95				

a. R Squared = .736 (Adjusted R Squared = .728)

B

Post Hoc Tests

Multiple Comparisons

Dependent Variable: CXCL12vivo_ELISA

Bonferroni

(I) Program	(J) Program	Mean Difference (I-J)	Std. Error	Sig.	95% Confidence Interval Lower Bound	95% Confidence Interval Upper Bound
1.00	2.00	-16.6728*	1.32577	.000	-20.2477	-13.0978
	3.00	-15.7433*	1.32577	.000	-19.3183	-12.1684
	4.00	-2.6146	1.32577	.310	-6.1895	.9604
2.00	1.00	16.6728*	1.32577	.000	13.0978	20.2477
	3.00	.9295	1.32577	1.000	-2.6455	4.5044
	4.00	14.0582*	1.32577	.000	10.4833	17.6332
3.00	1.00	15.7433*	1.32577	.000	12.1684	19.3183
	2.00	-.9295	1.32577	1.000	-4.5044	2.6455
	4.00	13.1288*	1.32577	.000	9.5538	16.7037
4.00	1.00	2.6146	1.32577	.310	-.9604	6.1895
	2.00	-14.0582*	1.32577	.000	-17.6332	-10.4833
	3.00	-13.1288*	1.32577	.000	-16.7037	-9.5538

Based on observed means.
The error term is Mean Square(Error) = 21.092.

*. The mean difference is significant at the .05 level.

Program (= different treatement)

1.00 = Naive mice
2.00 = Tumor-bearing mice
3.00 = Tumor-bearing mice + Vehicle
4.00 = Tumor-bearing mice + Imatinib

A

Tests of Between-Subjects Effects

Dependent Variable: S100A4vivo_ELISA

Source	Type III Sum of Squares	df	Mean Square	F	Sig.	Partial Eta Squared
Corrected Model	3856.936 [a]	3	1285.645	106.049	.000	.776
Intercept	9225.603	1	9225.603	760.991	.000	.892
Program	3856.936	3	1285.645	106.049	.000	.776
Error	1115.329	92	12.123			
Total	14197.868	96				
Corrected Total	4972.265	95				

a. R Squared = .776 (Adjusted R Squared = .768)

B

Post Hoc Tests

Multiple Comparisons

Dependent Variable: S100A4vivo_ELISA
Bonferroni

(I) Program	(J) Program	Mean Difference (I-J)	Std. Error	Sig.	95% Confidence Interval	
					Lower Bound	Upper Bound
1.00	2.00	-14.0908*	1.00512	.000	-16.8011	-11.3805
	3.00	-15.0117*	1.00512	.000	-17.7220	-12.3014
	4.00	-4.6514*	1.00512	.000	-7.3617	-1.9411
2.00	1.00	14.0908*	1.00512	.000	11.3805	16.8011
	3.00	-.9208	1.00512	1.000	-3.6311	1.7895
	4.00	9.4394*	1.00512	.000	6.7291	12.1497
3.00	1.00	15.0117*	1.00512	.000	12.3014	17.7220
	2.00	.9208	1.00512	1.000	-1.7895	3.6311
	4.00	10.3602*	1.00512	.000	7.6499	13.0706
4.00	1.00	4.6514*	1.00512	.000	1.9411	7.3617
	2.00	-9.4394*	1.00512	.000	-12.1497	-6.7291
	3.00	-10.3602*	1.00512	.000	-13.0706	-7.6499

Based on observed means.
The error term is Mean Square(Error) = 12.123.

*. The mean difference is significant at the .05 level.

Program (= different treatement)

1.00 = Naive mice

2.00 = Tumor-bearing mice

3.00 = Tumor-bearing mice + Vehicle

4.00 = Tumor-bearing mice + Imatinib

A

Tests of Between-Subjects Effects

Dependent Variable: CCL2vivo_ELISA

Source	Type III Sum of Squares	df	Mean Square	F	Sig.	Partial Eta Squared
Corrected Model	133.152[a]	5	26.630	214.125	.000	.904
Intercept	101.845	1	101.845	818.897	.000	.878
Program	133.152	5	26.630	214.125	.000	.904
Error	14.178	114	.124			
Total	310.612	120				
Corrected Total	147.330	119				

a. R Squared = .904 (Adjusted R Squared = .900)

B

Post Hoc Tests

Multiple Comparisons

Dependent Variable: CCL2vivo_ELISA

Bonferroni

(I) Program	(J) Program	Mean Difference (I-J)	Std. Error	Sig.	95% Confidence Interval Lower Bound	95% Confidence Interval Upper Bound
1.00	2.00	-2.3062*	.07886	.000	-2.5426	-2.0697
	3.00	-2.3664*	.12468	.000	-2.7403	-1.9926
	4.00	-.1554	.12468	1.000	-.5293	.2184
	5.00	-.7910*	.12468	.000	-1.1649	-.4172
	6.00	-.8413*	.12468	.000	-1.2152	-.4675
2.00	1.00	2.3062*	.07886	.000	2.0697	2.5426
	3.00	-.0602	.12468	1.000	-.4341	.3136
	4.00	2.1508*	.12468	.000	1.7769	2.5246
	5.00	1.5151*	.12468	.000	1.1413	1.8890
	6.00	1.4648*	.12468	.000	1.0910	1.8387
3.00	1.00	2.3664*	.12468	.000	1.9926	2.7403
	2.00	.0602	.12468	1.000	-.3136	.4341
	4.00	2.2110*	.15771	.000	1.7381	2.6839
	5.00	1.5754*	.15771	.000	1.1025	2.0483
	6.00	1.5251*	.15771	.000	1.0522	1.9980
4.00	1.00	.1554	.12468	1.000	-.2184	.5293
	2.00	-2.1507*	.12468	.000	-2.5246	-1.7769
	3.00	-2.2110*	.15771	.000	-2.6839	-1.7381
	5.00	-.6356*	.15771	.002	-1.1085	-.1627
	6.00	-.6859*	.15771	.000	-1.1588	-.2130
5.00	1.00	.7910*	.12468	.000	.4172	1.1649
	2.00	-1.5151*	.12468	.000	-1.8890	-1.1413
	3.00	-1.5754*	.15771	.000	-2.0483	-1.1025
	4.00	.6356*	.15771	.002	.1627	1.1085
	6.00	-.0503	.15771	1.000	-.5232	.4226
6.00	1.00	.8413*	.12468	.000	.4675	1.2152
	2.00	-1.4648*	.12468	.000	-1.8387	-1.0910
	3.00	-1.5251*	.15771	.000	-1.9980	-1.0522
	4.00	.6859*	.15771	.000	.2130	1.1588
	5.00	.0503	.15771	1.000	-.4226	.5232

Based on observed means.
The error term is Mean Square(Error) = .124.

*. The mean difference is significant at the .05 level.

Program (= different treatement)

1.00 = Naive mice
2.00 = Tumor-bearing mice
3.00 = Tumor-bearing mice + Vehicle
4.00 = Tumor-bearing mice + Imatinib
5.00 = Tumor-bearing mice + FPS-ZM1
6.00 =Tumor-bearing mice + TAK-242

A

Tests of Between-Subjects Effects

Dependent Variable: Gas6vivo_ELISA

Source	Type III Sum of Squares	df	Mean Square	F	Sig.	Partial Eta Squared
Corrected Model	25538.402 [a]	5	5107.680	273.619	.000	.923
Intercept	21285.137	1	21285.137	1140.245	.000	.909
Program	25538.402	5	5107.680	273.619	.000	.923
Error	2128.056	114	18.667			
Total	62369.106	120				
Corrected Total	27666.458	119				

a. R Squared = .923 (Adjusted R Squared = .920)

B

Post Hoc Tests

Multiple Comparisons

Dependent Variable: Gas6vivo_ELISA

Bonferroni

(I) Program	(J) Program	Mean Difference (I-J)	Std. Error	Sig.	95% Confidence Interval Lower Bound	Upper Bound
1.00	2.00	-31.6702*	.96610	.000	-34.5669	-28.7736
	3.00	-33.6405*	1.52755	.000	-38.2206	-29.0604
	4.00	-4.7195*	1.52755	.038	-9.2996	-.1394
	5.00	-7.9771*	1.52755	.000	-12.5572	-3.3970
	6.00	-8.6745*	1.52755	.000	-13.2546	-4.0944
2.00	1.00	31.6703*	.96610	.000	28.7736	34.5669
	3.00	-1.9702	1.52755	1.000	-6.5503	2.6098
	4.00	26.9508*	1.52755	.000	22.3707	31.5308
	5.00	23.6932*	1.52755	.000	19.1131	28.2732
	6.00	22.9958*	1.52755	.000	18.4157	27.5758
3.00	1.00	33.6405*	1.52755	.000	29.0604	38.2206
	2.00	1.9703	1.52755	1.000	-2.6098	6.5503
	4.00	28.9210*	1.93221	.000	23.1276	34.7144
	5.00	25.6634*	1.93221	.000	19.8700	31.4568
	6.00	24.9660*	1.93221	.000	19.1726	30.7594
4.00	1.00	4.7195*	1.52755	.038	.1394	9.2996
	2.00	-26.9507*	1.52755	.000	-31.5308	-22.3707
	3.00	-28.9210*	1.93221	.000	-34.7144	-23.1276
	5.00	-3.2576	1.93221	1.000	-9.0510	2.5358
	6.00	-3.9550	1.93221	.645	-9.7484	1.8384
5.00	1.00	7.9771*	1.52755	.000	3.3970	12.5572
	2.00	-23.6932*	1.52755	.000	-28.2732	-19.1131
	3.00	-25.6634*	1.93221	.000	-31.4568	-19.8700
	4.00	3.2576	1.93221	1.000	-2.5358	9.0510
	6.00	-.6974	1.93221	1.000	-6.4908	5.0960
6.00	1.00	8.6745*	1.52755	.000	4.0944	13.2546
	2.00	-22.9957*	1.52755	.000	-27.5758	-18.4157
	3.00	-24.9660*	1.93221	.000	-30.7594	-19.1726
	4.00	3.9550	1.93221	.645	-1.8384	9.7484
	5.00	.6974	1.93221	1.000	-5.0960	6.4908

Based on observed means.
The error term is Mean Square(Error) = 18.667.
*. The mean difference is significant at the .05 level.

Program (= different treatement)

1.00 = Naive mice
2.00 = Tumor-bearing mice
3.00 = Tumor-bearing mice + Vehicle
4.00 = Tumor-bearing mice + Imatinib
5.00 = Tumor-bearing mice + FPS-ZM1
6.00 =Tumor-bearing mice + TAK-242

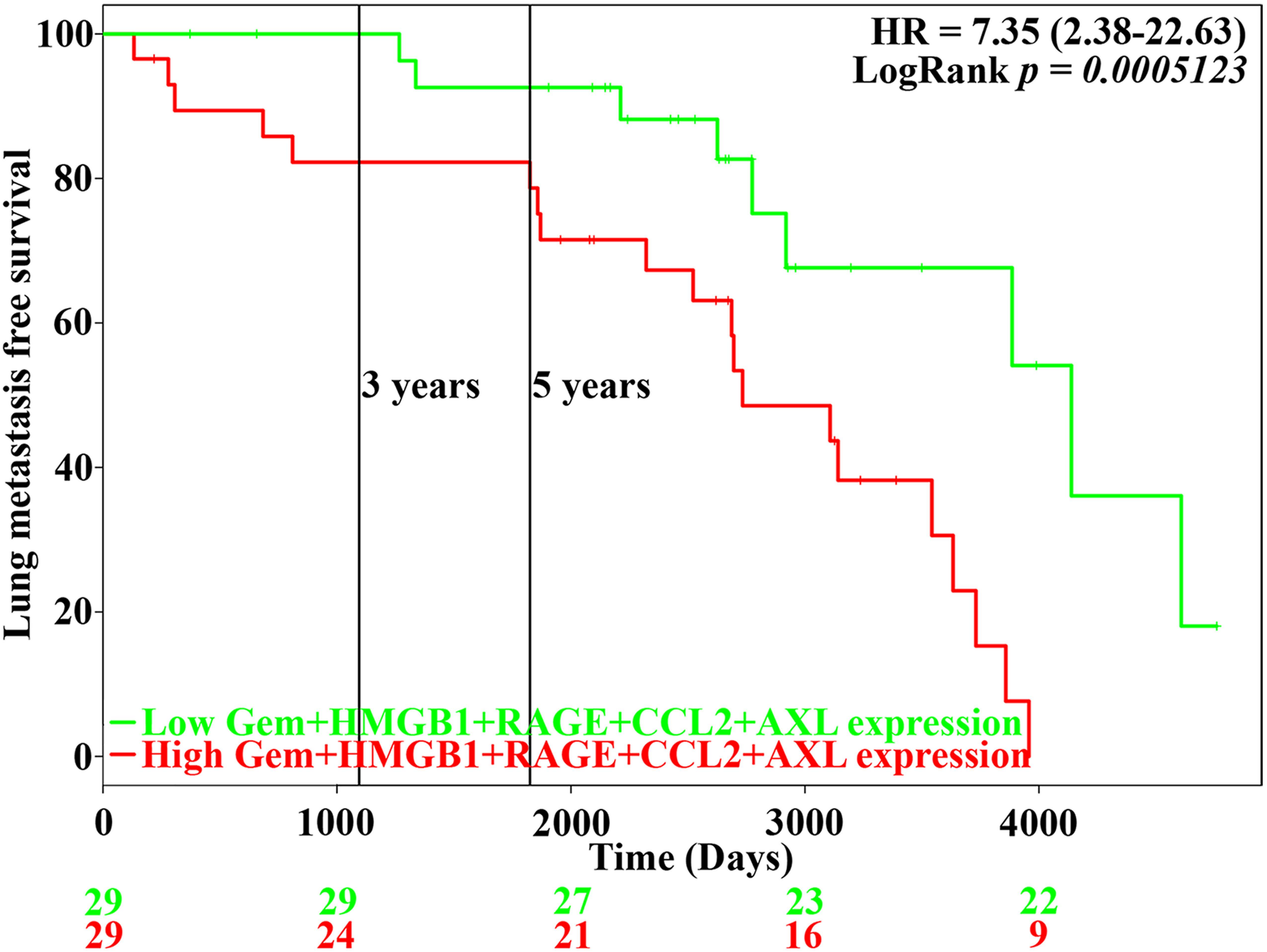
HR = 7.35 (2.38-22.63)
LogRank p = 0.0005123
Lung metastasis free survival
100
80
60
40
20
0
3 years
5 years
Low Gem+HMGB1+RAGE+CCL2+AXL expression
High Gem+HMGB1+RAGE+CCL2+AXL expression
0
1000
2000
3000
4000
Time (Days)
29 29 27 23 22
29 24 21 16 9

A

Tests of Between-Subjects Effects

Dependent Variable: G240_Invasion

Source	Type III Sum of Squares	df	Mean Square	F	Sig.	Partial Eta Squared
Corrected Model	5386461.9 [a]	4	1346615.48	320.697	.000	.970
Intercept	17957651.8	1	17957651.8	4276.616	.000	.991
Program	5386461.91	4	1346615.48	320.697	.000	.970
Error	167961.333	40	4199.033			
Total	23512075.0	45				
Corrected Total	5554423.24	44				

a. R Squared = .970 (Adjusted R Squared = .967)

B

Post Hoc Tests

Multiple Comparisons

Dependent Variable: G240_Invasion
Bonferroni

(I) Program	(J) Program	Mean Difference (I-J)	Std. Error	Sig.	95% Confidence Interval Lower Bound	95% Confidence Interval Upper Bound
1.00	2.00	-845.8889*	30.54699	.000	-936.6492	-755.1286
	3.00	-45.8889	30.54699	1.000	-136.6492	44.8714
	4.00	101.5556*	30.54699	.019	10.7952	192.3159
	5.00	-15.0000	30.54699	1.000	-105.7603	75.7603
2.00	1.00	845.8889*	30.54699	.000	755.1286	936.6492
	3.00	800.0000*	30.54699	.000	709.2397	890.7603
	4.00	947.4444*	30.54699	.000	856.6841	1038.2048
	5.00	830.8889*	30.54699	.000	740.1286	921.6492
3.00	1.00	45.8889	30.54699	1.000	-44.8714	136.6492
	2.00	-800.0000*	30.54699	.000	-890.7603	-709.2397
	4.00	147.4444*	30.54699	.000	56.6841	238.2048
	5.00	30.8889	30.54699	1.000	-59.8714	121.6492
4.00	1.00	-101.5556*	30.54699	.019	-192.3159	-10.7952
	2.00	-947.4444*	30.54699	.000	-1038.2048	-856.6841
	3.00	-147.4444*	30.54699	.000	-238.2048	-56.6841
	5.00	-116.5556*	30.54699	.005	-207.3159	-25.7952
5.00	1.00	15.0000	30.54699	1.000	-75.7603	105.7603
	2.00	-830.8889*	30.54699	.000	-921.6492	-740.1286
	3.00	-30.8889	30.54699	1.000	-121.6492	59.8714
	4.00	116.5556*	30.54699	.005	25.7952	207.3159

Based on observed means.
The error term is Mean Square(Error) = 4199.033.

*. The mean difference is significant at the .05 level.

Program (= different treatement)

1.00 = Serum-free (SF) medium
2.00 = SF + S100A4/Gas6 + Vehicle
3.00 = SF + S100A4/Gas6 + FPS-ZM1
4.00 = SF + S100A4/Gas6 + R428
5.00 = SF + S100A4/Gas6 + FPS-ZM1/R428

A

Tests of Between-Subjects Effects

Dependent Variable: G257_Invasion

Source	Type III Sum of Squares	df	Mean Square	F	Sig.	Partial Eta Squared
Corrected Model	15261525[a]	4	3815381.26	414.637	.000	.976
Intercept	67866648.2	1	67866648.2	7375.411	.000	.995
Program	15261525.0	4	3815381.26	414.637	.000	.976
Error	368069.778	40	9201.744			
Total	83496243.0	45				
Corrected Total	15629594.8	44				

a. R Squared = .976 (Adjusted R Squared = .974)

B

Post Hoc Tests

Multiple Comparisons

Dependent Variable: G257_Inavsion

Bonferroni

(I) Program	(J) Program	Mean Difference (I-J)	Std. Error	Sig.	95% Confidence Interval Lower Bound	95% Confidence Interval Upper Bound
1.00	2.00	-370.0000*	45.21982	.000	-504.3558	-235.6442
	3.00	-389.0000*	45.21982	.000	-523.3558	-254.6442
	4.00	970.7778*	45.21982	.000	836.4219	1105.1336
	5.00	824.5556*	45.21982	.000	690.1997	958.9114
2.00	1.00	370.0000*	45.21982	.000	235.6442	504.3558
	3.00	-19.0000	45.21982	1.000	-153.3558	115.3558
	4.00	1340.7778*	45.21982	.000	1206.4219	1475.1336
	5.00	1194.5556*	45.21982	.000	1060.1997	1328.9114
3.00	1.00	389.0000*	45.21982	.000	254.6442	523.3558
	2.00	19.0000	45.21982	1.000	-115.3558	153.3558
	4.00	1359.7778*	45.21982	.000	1225.4219	1494.1336
	5.00	1213.5556*	45.21982	.000	1079.1997	1347.9114
4.00	1.00	-970.7778*	45.21982	.000	-1105.1336	-836.4219
	2.00	-1340.778*	45.21982	.000	-1475.1336	-1206.4219
	3.00	-1359.778*	45.21982	.000	-1494.1336	-1225.4219
	5.00	-146.2222*	45.21982	.025	-280.5781	-11.8664
5.00	1.00	-824.5556*	45.21982	.000	-958.9114	-690.1997
	2.00	-1194.556*	45.21982	.000	-1328.9114	-1060.1997
	3.00	-1213.556*	45.21982	.000	-1347.9114	-1079.1997
	4.00	146.2222*	45.21982	.025	11.8664	280.5781

Based on observed means.
The error term is Mean Square(Error) = 9201.744.

*. The mean difference is significant at the .05 level.

Program (= different treatement)

1.00 = Serum-free (SF) medium
2.00 = SF + S100A4/Gas6 + Vehicle
3.00 = SF + S100A4/Gas6 + FPS-ZM1
4.00 = SF + S100A4/Gas6 + R428
5.00 = SF + S100A4/Gas6 + FPS-ZM1/R428

A

Tests of Between-Subjects Effects

Dependent Variable: Matrix_Binding

Source	Type III Sum of Squares	df	Mean Square	F	Sig.
Corrected Model	618638.39[a]	4	154659.597	20.968	.000
Intercept	9175608.89	1	9175608.89	1243.969	.000
Program	618638.389	4	154659.597	20.968	.000
Error	1290812.72	175	7376.073		
Total	11085060.0	180			
Corrected Total	1909451.11	179			

a. R Squared = .324 (Adjusted R Squared = .309)

B

Post Hoc Tests

Multiple Comparisons

Dependent Variable: Matrix_Binding

Bonferroni

(I) Program	(J) Program	Mean Difference (I-J)	Std. Error	Sig.	95% Confidence Interval Lower Bound	95% Confidence Interval Upper Bound
1.00	2.00	-113.5000*	20.24307	.000	-171.0523	-55.9477
	3.00	-1.5000	20.24307	1.000	-59.0523	56.0523
	4.00	44.5833	20.24307	.289	-12.9690	102.1357
	5.00	49.0278	20.24307	.165	-8.5246	106.5801
2.00	1.00	113.5000*	20.24307	.000	55.9477	171.0523
	3.00	112.0000*	20.24307	.000	54.4477	169.5523
	4.00	158.0833*	20.24307	.000	100.5310	215.6357
	5.00	162.5278*	20.24307	.000	104.9754	220.0801
3.00	1.00	1.5000	20.24307	1.000	-56.0523	59.0523
	2.00	-112.0000*	20.24307	.000	-169.5523	-54.4477
	4.00	46.0833	20.24307	.240	-11.4690	103.6357
	5.00	50.5278	20.24307	.135	-7.0246	108.0801
4.00	1.00	-44.5833	20.24307	.289	-102.1357	12.9690
	2.00	-158.0833*	20.24307	.000	-215.6357	-100.5310
	3.00	-46.0833	20.24307	.240	-103.6357	11.4690
	5.00	4.4444	20.24307	1.000	-53.1079	61.9968
5.00	1.00	-49.0278	20.24307	.165	-106.5801	8.5246
	2.00	-162.5278*	20.24307	.000	-220.0801	-104.9754
	3.00	-50.5278	20.24307	.135	-108.0801	7.0246
	4.00	-4.4444	20.24307	1.000	-61.9968	53.1079

Based on observed means.
The error term is Mean Square(Error) = 7376.073.
*. The mean difference is significant at the 0.05 level.

Program

1.00 = Serum-free (SF) medium
2.00 = SF + S100A4/Gas6 + Vehicle
3.00 = SF + S100A4/Gas6 + FPS-ZM1
4.00 = SF + S100A4/Gas6 + R428
5.00 = SF + S100A4/Gas6 + FPS-ZM1/R428

Supplemental Figure 1. Dox-dependent HMGB1 secretion. The level of HMGB1 secreted from the indicated cell lines following doxycycline treatment and under normoxic or hypoxic conditions. Assay performed 3 separate times each in triplicates.

Supplemental Figure 2. ANOVA test followed by a post hoc Bonferroni test for HMGB1 secretion from HME, Gem240, and Gem257 cells grown under normoxia or hypoxia in the presence of vehicle or imatinib for 24h. Related to Fig. 1A.

Supplemental Figure 3. GemOE CM induces oncogenic S100 mRNAs expression in MSCs. (A) The expression level of the indicated *mRNA* normalized to *GAPDH mRNA* in MSCs exposed to CM from indicated cell lines for 24h. Total *RNA* was isolated and assessed for the expression of these *mRNAs* using real-time RT/PCR. Each assay was performed 3 separate times each in triplicates. (B) The expression level of the indicated *mRNA* normalized to *GAPDH mRNA* in MSCs exposed to CM from indicated cell lines for 24h in the presence of IgG or HMGB1 NeuAb. Total *RNA* was isolated and assessed for the expression of these *mRNAs* using real-time RT/PCR. Each assay was performed 3 separate times each in triplicates.

Supplemental Figure 4. ANOVA test followed by a post hoc Bonferroni test for S100A4 secretion from MSCs exposed to CM from HME, Gem240, and Gem257 cells grown under normoxia or hypoxia in the presence of vehicle or anti-HMGB1 NeuAb. For 24h. Related to Fig. 1F.

Supplemental Figure 5. ANOVA test followed by a post hoc Bonferroni test for CCL2 secretion from HME, Gem240, and Gem257 cells exposed to CM from the same cell/MSCs co-cultures grown under normoxia or hypoxia in the presence of vehicles or anti-S100A4 NeuAb. or Glycyrrhizin for 24h. Related to Fig. 2B.

Supplemental Figure 6. ANOVA test followed by a post hoc Bonferroni test for Gas6 secretion from THP1s exposed to CM from HME/MSCs, Gem240/MSCs or Gem257/MSCs co-cultures recondition by HME, Gem240, or Gem257 cells, respectively, grown under normoxia or hypoxia in the presence of vehicles or CCL2 NeuAb. or BMS CCR2 22 for 24h. Related to Fig. 2G.

Supplemental Figure 7. ANOVA test followed by a post hoc Bonferroni test for HMGB1 in sera from naïve mice, Gem240, or Gem257 tumors-bearing mice treated with None, Vehicle, or Imatinib as shown in Fig. 3A. Related to Fig. 3D.

Supplemental Figure 8. ANOVA test followed by a post hoc Bonferroni test for CXCL12 in sera from naïve mice, Gem240, or Gem257 tumors-bearing mice treated with None, Vehicle, or Imatinib as shown in Fig. 3A. Related to Fig. 3E.

Supplemental Figure 9. ANOVA test followed by a post hoc Bonferroni test for S100A4 in sera from naïve mice, Gem240, or Gem257 tumors-bearing mice treated with None, Vehicle, or Imatinib as shown in Fig. 3A. Related to Fig. 3F.

Supplemental Figure 10. ANOVA test followed by a post hoc Bonferroni test for CCL2 in sera from naïve mice, Gem240, or Gem257 tumors-bearing mice treated with None, Vehicle, Imatinib, FPS-ZM1, or TAK-242 as shown in Fig. 4A. Related to Fig. 4E, upper.

Supplemental Figure 11. ANOVA test followed by a post hoc Bonferroni test for Gas6in sera from naïve mice, Gem240, or Gem257 tumors-bearing mice treated with None, Vehicle, Imatinib, FPS-ZM1, or TAK-242 as shown in Fig. 4A. Related to Fig. 4E, lower.

Supplemental Figure 12. Further evidence from human data for the positive feedback loops-induced lung metastasis. Kaplan Meir analysis of lung metastasis-free survival in geminin+HMGB1+RAGE+CCL2+AXL overexpressing patients vs. low expressing patients.

Supplemental Figure 13. ANOVA test followed by a post hoc Bonferroni test for Gem240 cells invasion of Matrigel in Serum-free (SF) medium (none), or SF-medium containing rS100A4+rGas6 *plus* Vehicle, FPS-ZM1, R428, or FPS-ZM1+R428. Related to Fig. 6C and 6D.

Supplemental Figure 14. ANOVA test followed by a post hoc Bonferroni test for Gem257 cells invasion of Matrigel in Serum-free (SF) medium (none), or SF-medium containing rS100A4+rGas6 *plus* Vehicle, FPS-ZM1, R428, or FPS-ZM1+R428. Related to Fig. 6C and 6D.

Supplemental Figure 15. ANOVA test followed by a post hoc Bonferroni test for Gem240 cells binding to plastic, or Laminin I, Collagen IV, or Fibronectin conducted in SF-medium (none), or SF-medium containing rS100A4+rGas6 *plus* Vehicle, FPS-ZM1, R428, or FPS-ZM1+R428. Related to Fig. 6C and 6D. Identical results were obtained for Gem257 cells.
